# DeepH-DTA: Deep Learning for Predicting Drug-Target Interactions: A Case Study of COVID-19 Drug Repurposing

**DOI:** 10.1109/ACCESS.2020.3024238

**Published:** 2020-09-15

**Authors:** Mohamed Abdel-Basset, Hossam Hawash, Mohamed Elhoseny, Ripon K. Chakrabortty, Michael Ryan

**Affiliations:** 1 Faculty of Computers and InformaticsZagazig University 68799 Zagazig 44519 Egypt; 2 Department of Computer ScienceCollege of Computer Information TechnologyAmerican University in the Emirates 219929 Dubai 503000 United Arab Emirates; 3 Faculty of Computers and InformationMansoura University 68779 Mansoura 35516 Egypt; 4 Capability Systems Centre, School of Engineering and ITUniversity of New South Wales Canberra Canberra ACT 2612 Australia

**Keywords:** Deep learning, drug-target interaction, SARS-CoV-2

## Abstract

The rapid spread of novel coronavirus pneumonia (COVID-19) has led to a dramatically increased mortality rate worldwide. Despite many efforts, the rapid development of an effective vaccine for this novel virus will take considerable time and relies on the identification of drug-target (DT) interactions utilizing commercially available medication to identify potential inhibitors. Motivated by this, we propose a new framework, called DeepH-DTA, for predicting DT binding affinities for heterogeneous drugs. We propose a heterogeneous graph attention (HGAT) model to learn topological information of compound molecules and bidirectional ConvLSTM layers for modeling spatio-sequential information in simplified molecular-input line-entry system (SMILES) sequences of drug data. For protein sequences, we propose a squeezed-excited dense convolutional network for learning hidden representations within amino acid sequences; while utilizing advanced embedding techniques for encoding both kinds of input sequences. The performance of DeepH-DTA is evaluated through extensive experiments against cutting-edge approaches utilising two public datasets (Davis, and KIBA) which comprise eclectic samples of the kinase protein family and the pertinent inhibitors. DeepH-DTA attains the highest Concordance Index (CI) of 0.924 and 0.927 and also achieved a mean square error (MSE) of 0.195 and 0.111 on the Davis and KIBA datasets respectively. Moreover, a study using FDA-approved drugs from the Drug Bank database is performed using DeepH-DTA to predict the affinity scores of drugs against SARS-CoV-2 amino acid sequences, and the results show that that the model can predict some of the SARS-Cov-2 inhibitors that have been recently approved in many clinical studies.

## I. Introduction

One of the preliminary stages of drug discovery is the determination of innovative candidate drug compounds that interact with particular target proteins. Through in vivo and in vitro studies, several high-throughput experiments have been conducted to identify the novel compounds with the anticipated interactive characteristics [1]. However, expensive costs and chronological order requirements make it impracticable to scan immense volumes of targets and mixtures. Consequently, the identification of novel drugs takes an extraordinarily long time [2].

At present, the compound database (i.e., PubChem, ChEMBL) contains over 105 million compound candidates, and more than 250 million bioactivities in both data sets (combined) [3], [4]. On the other hand, the recent number of FDA-approved drugs is about 10000, according to DrugBank [5]. Additionally, only a small number of proteins in the human proteome are targeted by recognized drugs. According to current statistics, knowledge of the drug–target (DT) space is still incomplete and requires a novel approach to enable broader investigation [8].

### A. Research Motivation

Recognizing drug-target interactions (DTI) is a critical phase in the process of discovering and developing new drugs that enable the repurposing of prevailing drugs and singles out the novel interactive partners for approved drugs. Consequently, DTI has attracted much research attention.

Until recently, the task of modeling DTI has been addressed as a binary classification problem ignoring a vitally significant section of characteristics regarding protein-ligand interactions, specifically the binding affinity scores which represent interactivity strength between DT pairs. Such scores are regularly quantified with measures such as half-maximal inhibitory concentration (IC50) which relies on the attentiveness of the ligand and target, dissociation constant ($K_{d}$), and inhibition constant ($K_{i}$) [6]. Lower values of IC50, $K_{i}\mathrm {, }$ and $K_{d}$ indicate strong binding affinity. $K_{d}$ and $K_{i}$ values are typically used to compute the negative logarithm of the dissociation or inhibition constants and denoted as $pK_{d}$ or $pK_{i}$ [7], [29]. In DTI binary classification studies, dataset construction is a significant stage, since the selection of the non-binding instances directly influences the performance of the model [6], [8], [10]. Recently, four datasets have been widely used in several DTI studies in which pairs of DTs with unknown binding evidence are considered as non-binding instances. Recently, DTI studies that depend on affinity information databases have offered a new representative binary dataset formed with a selected binding affinity threshold value.

As explained in [7], [11], [12], [27], [30], treating DTI as a binary classification problem has two main drawbacks. First, the true-negative interactions and unidentified scores that are not discriminated against. Second, the binary associations are broadly known to be very intelligible, while it is more instructive to harness a continuous value that estimates the binding strength between a drug molecule and the target sequences which is articulated in terms of the beforementioned measures. Accordingly, researchers have been motivated to address the DTI as a regression problem. This, in turn, offers a number of advantages. First, it avoids the impact of selecting a negative sample on the deep learning approach and can deliver additional applied and valuable information [27]. Second, it allows the development of more accurate models, as well as the construction of a more realistic database. Third, a regression-based model has the benefit of forecasting an approximate value of the strength of the DT interaction which, in turn, can be significantly advantageous for the reduction of the enormous compound search-space in the process of drug discovery.

In early DTI systems, conventional machine-learning (ML) approaches have been utilized, such as support vector machine (SVM) and naïve Bayesian (NB) [9]. The performances of these approaches primarily depend on the surface-level features captured from drug data and protein sequences. However, adding additional shallow features does not lead to increase performance because of the probable intensification of features. Therefore, to guarantee the effective recognition of compound-target interactions, the typical procedure is to extract a vast quantity of such shallow features ignoring whether they are finally exploited for identification of interactions, and then feature selection techniques—such as Principle Component Analysis (PCA)—are adapted to form the critical DT features into a uniform vector space [6]. Such traditional learning-based DTI schemes, however, are unable to perform well for modeling complex interactions. Currently, deep learning-based DTI models have gained increased attention due to their ability to automatically learn and extract feature representations through the numerous internal hidden layers [28], [29].

As a result of remarkable performance in such applications as computer visionand speech synthesis, deep learning has become widely used in bioinformatics as well as in quantitative structure-activity relationship (QSAR) studies in drug discovery [1], making use of efficient data representations using non-linear transformations that smooth the learning process of embedded hidden patterns. A small number of studies adopted deep neural networks (DNN) for predicting DTI binary class employing various inputs of proteins and drugs. In particular, the convolutional neural network (CNN) architectures are broadly utilized for modeling DTI characteristics [7]. Despite their advantages, CNN-based approaches are inefficient in that they only capture invariant local patterns and do not capture the long-term dependencies [10]. Extracting the global information of protein sequences and drug compounds will not only improve the efficiency of DTI but will also support the detection of complex interactions. Recurrent neural network (RNN) architectures are proposed for sequential data, where the current data element state is calculated depending on the preceding one or the upcoming one. Mainly, long short-term memory (LSTM) models are talented to capture and remember longer sequences compared to simple RNN or Gated Recurrent Unit (GRUs) which make it the best choice to learn sequential dependencies within molecule sequences or amino acid sequences [6].

### B. SARS-COV-2

Since late December 2019, human beings across the world continents have been subject to viral infection and mass transmission of a novel coronavirus that has caused widespread infection in birds, mammals, and humans [15]. The virus was identified as non-partitioned positive-strand ribonucleic acid (RNA) belonging to the Coronavirinae species. Scientifically, it is called severe acute respiratory syndrome coronavirus 2 (SARS-COV-2) [16]. The Coronavirinae species primarily comprises four genera: Alphacoronavirus, Betacoronavirus, Deltacoronavirus, and Gammacoronavirus [17]. The Betacoronavirus genus contains two notorious infectious coronaviruses namely: the Middle East respiratory syndrome coronavirus (MERS-CoV) [18] and severe acute respiratory syndrome coronavirus (SARS-CoV), which have recorded a lot of infection cases that exceed 10,000 cases, with death rates of 37% and 10% respectively [19]. Such rapid infection rates lead to an urgent demand for treatment to inhibit, if not prohibit, SARS-CoV2 prevalence [17]–[19]. Unfortunately, contemporary drug development cannot accomplish this task with sufficient speed and considerable time is required to develop a new drug and deliver it to the market. Such delays leave the world facing very high death rates due to the recent uncommon pneumonia identified as coronavirus disease 2019 (COVID-19) caused by SARS-CoV-2 [20].

SARS-CoV-2 is clinically identified as single-stranded RNA that belongs to Betacoronavirus. It encompasses genes encoding 3C-like proteinase (3Cpro), 2-O-ribose methyltransferase (2OMT), RNA-dependent RNA polymerase (RdRp), nucleocapsid phosphoprotein, envelope protein, spike protein, nucleocapsid phosphoprotein, and many other proteins, depending on the obtained genome sequences of SARS-CoV-2 [21]. The standard clinical symptoms of COVID-19 include dry cough, high fever and fatigue [49]–[55]. The replication process of SARS-CoV-2 entails several phases following the host cell entrance: 1) the genomic RNA (gRNA) translated onto polyproteins; 2) transformation of polyproteins with viral 3Cpro into smaller replicase-transcriptase proteins; 3) replication of gRNA with the replicase-transcriptase complex that comprises of RdRp, helicase, 30-to-50 exonuclease, endoRNAse, and 2OMT; and 4) the viral components assembly. These replication-related proteins are considered the main targets of next-entry remediation drugs to stamp out viral replication [22]. Despite significant international efforts, there are no novel drugs or vaccines for the treatment of SARS-CoV-2. COVID-19 patients rely solely on their immune systems and any available, but less-effective, drugs.

### C. Main Contributions

In this study, we introduce a novel deep-learning-based DTI framework, called deepH-DTA, which geometrically exploits existing the topological structure of drug molecules as input features, along with the corresponding molecular fingerprints. Although several studies incorporated structural representations of molecules for predictive tasks under various settings for drug development or discovery, none investigated twofold prediction of chemical interactions between protein sequences of target and homogeneity of drug candidate compounds. To this end, we propose a novel DTI prediction framework that utilizes a HGAT [23] for efficient modeling of interactions of various targeted topological representation of drugs. Simultaneously, we introduce two layers of bidirectional ConvLSTM [24] to capture spatio-sequential characteristics of drug sequences encoded in a simplified molecular-input line-entry system (SMILES) format [25]. The spatio-sequential sequences capture both positional features of input SMILES and the long-term dependency representation within input sequences.

To address this shortcoming that LSTM is unable to capture the spatial correlation within long term sequences, which means that ConvLSTM is the best choice. We introduce an optimized and applied approach for predicting drug-target affinity with superior performance over cutting-edge studies.

*Case Study:* The proposed DeepH-DTA is applied to recognize the commercially available antiviral drugs that have the potential to act as a suppressor for the viral components of SARS-CoV-2. Furthermore, the aim is to enable our model to learn effectively the interactions between drugs and SARS-COV-2. We adopt a comprehensive set of commercially available antiviral drugs from different heterogeneity that could potentially hinder the reproduction cycle of SARS-CoV-2, providing guidance to scientists looking to develop an effective drug.

The remainder of the paper is structured as follows: Section 2, reviews the most relevant literature and associated works on the identification of potential inhibitors of SARS-COV-2. Illustrative explanation of the proposed frameworks, as well as construction principles, are presented in Section 3. The recommended experimental configurations, the dataset employed, and the results obtained in comparison with current studies are discussed in Section 4. Section 5 provides potential inhibitors for SARS-COV-2. Section 6 present the managerial implication of the current study. Section 7 presents some limitations of the current study. Finally, Section 6 draws some conclusions o and explains the intended future research directions.

## II. Related Work

### A. Drug-Target Interactions

Adopting deep learning for prediction drug-target affinities (DTA) has been a useful technique that plays a significant role in several vaccine discoveries and development problems. In [26], He *et al*. proposed a gradient boosting algorithm for estimating drug affinities based on the handcrafted features created from drugs and target information. Pahikkala *et al*. [27] introduced a Kronecker Regularized approach for minimizing a cost function using a similarity matrix calculated via the Pubchem clustering server. However, these approaches rely heavily on the nature of feature engineering adopted. In contrast, Tsubaki *et al*. [28] introduced a deep-learning approach for modeling DT, but did not exploit the topological structure of chemical molecules. In [29], Ozkirimli *et al*. introduced two-stream CNN for modeling DTI and predicting affinity scores; albeit employing traditional word embedding and ignoring contextual information in input sequences. In [7], Ozkirimli *et al*. introduce a methodology for predicting DT binding affinities using CNN over word representation of protein and compound sequences demonstrating that most essential binding information implanted in the protein domain. Zhao *et al*. [13] proposed a generative adversarial network (GAN) to learn beneficial patterns within labeled and unlabeled sequences and utilized convolutional regression to forecast binding affinity score. However, they did not address GAN training on a small dataset. Similarly, Zhao *et al*. [14] introduced a CNN architecture accompanied by attentional mechanisms to determine which protein sequences are more significant for a drug and which drug SMILES sequences are more vital for a protein during its affinity estimation. However, all of these approaches transform drug compounds into a corresponding string representation that is not an effective method to characterize molecules. Utilizing such strings leads to the loss of molecular structure information, which in turn could weaken the predictive performance of the model and the operational relevance of the learned hidden space.

On the other hand, Nguyen *et al*. [11] exploited the topological drug information through graph networks, namely graph convolutional network (GCN), graph attention network (GAN), and graph isomorphism network (GIN). Similarly, Wang *et al*. [12] utilized these networks to learn structural information of drug as well as protein dipeptide frequency of word frequency encoding to predict affinity score using different graph networks. Moreover, Lin *et al*. [30] proposed a novel deep learning approach for predicting drug-target affinities. However, these approaches use conventional CNN to process protein sequences which suffer from information losses in deeper layers and also unable to exploit the dependencies between various convolutional channels. More, they did not consider exploiting spatio-sequential information within SMILES sequences. The graph networks adopted in these studies did not consider the topological heterogeneity of drug molecules, which that they fail to generalize for heterogeneity-based applications.

### B. SARS-COV-2 Drug Repurposing

In addressing SARS-COV-2 inhibition, Beck *et al*. [31] adopted the pre-trained deep-molecule transformer architecture introduced in [32] depending on the mechanism of self-attention for identification of potential effective antiviral inhibitor against RdRp and 3Cpro of SARS-COV-2. However, they did not address the impact of SARS-COV or MERS-COV antiviral drugs on the SARS-COV-2. In [33], Hu *et al*. proposed a two-stage deep-learning model, where the shared layers were designated for joint representation modeling and the task-specific-layers were utilized for learning the weights of the specific blocks. However, they incorporated a small number of drugs in their experiments. Additionally, Ge *et al.* [34] introduced a data-driven approach that combines machine learning and statistical analysis for mining related disease data to predict the candidate’s antiviral inhibitor drug against SARS-CoV-2 by employing graph convolution model to exploit network topology data for capturing and calculating nodes’ pattern information to build a heterogeneous knowledge graph. In [35], Tang *et al*. proposed a novel fragment-based drug design architecture based on a new deep Q-learning network for producing and predicting lead compounds targeting 3CLpro of SARS-CoV-2. Besides, in [36], Zhavoronkov *et al*. employed three generative chemistry approaches for generating drug-like compounds (i.e., ligand-based generation, homology modelling-based generation, and crystal-derived pocked-based) as a potential antiviral for SARS-CoV-2. Nguyen *et al*. [37] showed that the binding sites corresponding to protease inhibitor of SARS-CoV-2 and SARS-CoV are very similar, and adopted a new deep generative network complex to learn affinity scores for pairs of drug-protein interaction to identify the optimal antiviral suppressors for COVID-19 spread. However, such a generative approach is computationally exhaustive, and the generated compounds have no clinical interpretation and require more biological investigation and optimization. Zhang *et al*. [38] adopted a full CNN for modeling protein-ligand interactions, conducting virtual screening for commercially existing drugs, and forecast the probable tripeptide lists for 3Cpro of SARS-CoV-2.

To sum up, the current studies of DTI can be divided into four groups: first, ML-based studies that often depend on feature engineering techniques; second, deep learning approaches that use the string representation of both drug and target which make them unable to capture the structure information of drug molecules; third, the graph networks used to learn drug representation within drugs from different nodes separately without addressing sequential characteristics of drug sequences. This in turn motivated us to propose a novel multi-path architecture that is able to learn both sequential and topological representation of input molecules, and also capture information from nodes and their connecting meta-paths.

## III. Methodology

**FIGURE 1.**
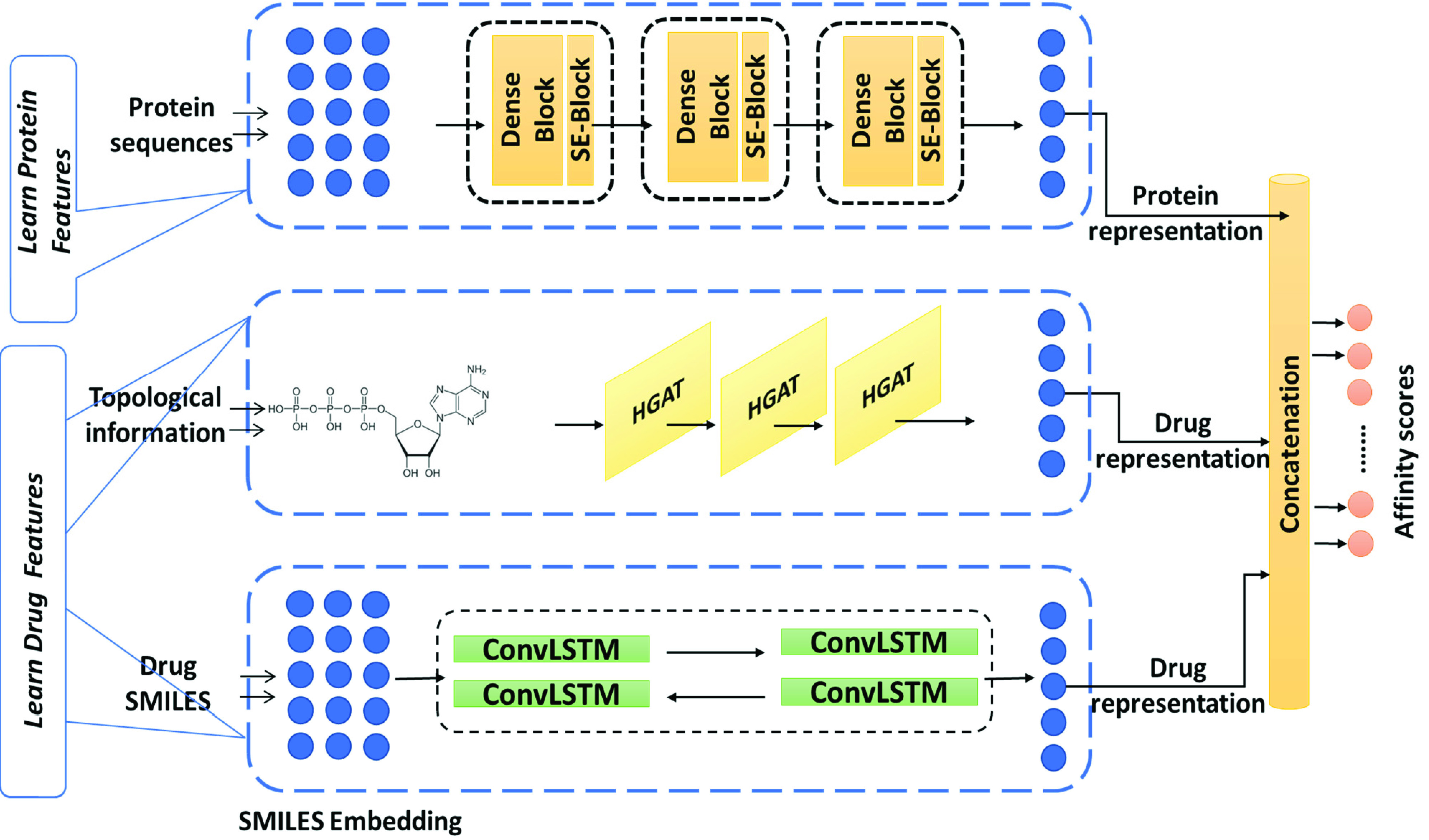
Illustration of the proposed DeepH-DTA. The upper module takes a fixed length of protein sequence as an input to produce corresponding protein information consisting of three dense blocks each followed by the SE block. The middle module processes the topological drug information to generate drug representation using improved heterogeneous GAT network. Concurrently, the lower module operates on the SMILES representations of drug to using Bidirectional ConvLSTM. Finally, the output of the three modules is concatenated to produce the final prediction score.

In this section we provide an explanation of our proposed three-channel approach called Deep heterogeneous learning framework for DTA (DeepH-DTA), and also introduce a detailed visualization of the proposed approach in Fig. 1. Our model primarily comprises four major blocks: (1) the first upper module is introduced to learn protein structure representation using Dense Net augmented with SE operation; (2) simultaneously, a heterogeneous graph network is introduced to learn the topological representation of drug molecules (the middle module Fig. 1); (3) the sequential characteristics of SMILES representation of input compounds is learned through bidirectional ConvLSTM architecture; and (4) the extracted representation is concatenated and fed into the output layer for affinity score calculation. The detailed explanation of the model’s implementation is discussed in the following subsections.

### A. Learn Target Sequences

In this section, amino acid sequences are encoded into embedding vector representation through the Polypeptide Frequency of Word Frequency approach [12] used for extracting protein features. Similar to bioinformatics calculation of term frequency (TF), the polypeptide frequency ($F$) can be computed with equation (1),

\begin{equation*} F={(v_{1},v_{2},v_{3}\cdots,v_{25^{n}})}^{T}\tag{1}\end{equation*}

where $v_{i}$ denotes the count of the $i$-th feature, and $n$ represents the numeral of 25 (as introduced in [12]) remains to exist in the polypeptide, hence ${25}^{n}$ diverse polymers are shaped by desiccation intensification. The calculation of $v_{i}$ is expressed in equation (2),

\begin{equation*} v_{i}=\frac {n^{u}}{\sum \nolimits _{u=1}^{25^{n}} n_{u}}=\frac {n_{u}}{L-1}\tag{2}\end{equation*}

where $n^{u}$ denotes the times of incidence of the $u$-th dipeptide pattern across the protein sequence, and $L$ is the protein sequence length. The inverse document frequency (IDF) calculated to raise the significant weight of TF is formulated in equation (3), where $N$ represents the number of protein sequences, and $W_{i}$ denotes the count of protein sequences incorporating the $i$-th polypeptide.

\begin{equation*} \mathrm {IDF}=\log {\left ({\frac {N}{W_{i}} }\right)\!,}\quad (1,2,3,\cdots,{25}^{n})\tag{3}\end{equation*}

Then the polypeptide frequency of word frequency can be calculated using equation (4),

\begin{equation*} \mathrm {WF} =({\mathrm {wf}}_{1},{\mathrm {wf}}_{2},{\mathrm {wf}}_{3}\cdots,{\mathrm {wf}}_{25^{ \mathrm {n}}}).\tag{4}\end{equation*}

where ${\mathrm {wf}}_{i}$ represents the frequency of the $i$-th polypeptide of word frequency and calculated with equation (5),

\begin{equation*} {\mathrm {wf}}_{i}=\frac {w_{i}}{N}\times \frac {p_{i}}{L-1}\tag{5}\end{equation*}

where $p_{i}$ represents the number of occurrences of the $i^{\mathrm {th}}$ peptide in the present protein sequence.

Subsequently, the concealed relationships corresponding to the polypeptide frequency of word frequency are attained through the proposed convolution model inspired by the DenseNet architecture [39], and three dense convolutional blocks are introduced to learn protein features. In each block, the collective knowledge of preceding convolutions is used as input for the current convolution, and a simple dropout layer is added after the first and the second layer of each block to avoid overfitting. Meanwhile, dense collective learning raises the channel count, so a transition is added between dense blocks to manage model complexity and to minimize the number of channels by using the $1\times 1$ convolutional layer.

*Squeeze and Excite (SE) Block:* Exploiting channel dependencies has been shown to enhance convolution model performance [40]. Thus, we attached spatial squeezing and channel excitation operations at the end of each block. Each of the $F$ filters convolve along their receptive field, which restrains the calculated convolutional output $X=[x_{1},\cdots,x_{F}]$ from making use of correlation information outside of this region, where $x_{f}\in \mathbb {R}^{W\times H}$ passed as input to squeeze and excite (SE) module to be combined using global average pooling (GAP) to produce a channel descriptor of the entire context of input channels. Hence, the spatial squeeze of the $f$-th channel is calculated using the spatial squeeze function $F_{\mathrm {sq}}$ as expressed in equation (6),

\begin{equation*} Z_{f}=F_{\mathrm {sq}}(x_{f})=\frac {1}{H\times W}\sum \limits _{i=1}^{H} \sum \limits _{j=1}^{w} {x_{f}(i,j)}\tag{6}\end{equation*}

where $x_{f}(i,j)$ denotes the spatial position of the $f$-th channel with width $W$ and height $H$. For clarification, the input feature map is compressed by GAP to yield $Z_{f}$.

An excitation operation is then applied to detect the channels’ nonlinear interaction and also to capture a non-mutually exclusive association using two fully connected layers (FCL), where the pooled vector of features is encoded to the dimension of $1\times 1\times \frac {F}{r}$, and then encoded to $1\times 1\times F$ using a simple gating operation with a sigmoid activation as formulated in equation (7),

\begin{equation*} s=F_{\mathrm {ex}}(Z,W\mathrm {) = \sigma (}W_{2}\& (W_{1}Z\mathrm {))}\tag{7}\end{equation*}

where & denotes the rectified linear unit function (Relu activation), $W_{1}\in {\mathbb {R}}^{F\times \frac {F}{r}}$, and $W_{2}\in {\mathbb {R}}^{F\times \frac {F}{r}}$ represents the parameters of the first FCL, respectively, and $r$ denotes reduction threshold used for complexity reduction and ease generalization; we achieved higher results with $r=2$. After that, a dimensionality-increasing layer is adopted in the second FCL to establish the dimension to the output’s channel. The output of the SE block is generated, the output U is computed and rescaled activations as:

\begin{equation*} \tilde {x}_{\mathrm {f}}=F_{\mathrm {scale}}(x_{f}\mathrm {,}{s}_{f}) =s_{f}\cdot x_{f}\tag{8}\end{equation*}

where $\tilde {X}_{f}= [x_{1}\mathrm {, }x_{2}\ldots \,\ldots \, x_{c}]$ and $F_{\mathrm {scale}}(x_{f},{s}_{c}\mathrm {)}$ represents the channel-wise production of feature maps $x_{f}\in \mathbb {R}^{H\times W}$ with the scalar value $s_{f}$.

### B. Learning Drug Features

#### 1) Topological Learning

A vital indication for the estimation of DTA is to effectively exploit molecular structure information to reveal the interconnection between atoms in the drug [11], [12]. Thus, to accomplish this, we transformed the SMILES chemical molecules molecule graph representation $G= (V,E)$, using RDKit^1^, where each node $v_{i}\in V$ denotes the $i$-th atom, and $e_{ij}\in E$ represents the chemical bond between the $i$-th and the $j$-th atoms. Graph attention networks (GAT) have shown their superiority for modeling graph representation in many studies [30]. However, it could be observed that the correlation between nodes in the generated compounds’ heterogeneous graph can have different semantics reflected in meta-paths, owing to the complication of the heterogeneous graph where every two objects (nodes) are linked via various semantic information paths, which are called meta-paths. Hence, adopting GAT is ineffective for such heterogeneous molecular graphs, since traditional GAT performs attention at the node level only and cannot exploit meta-path semantic relations. So, it cannot preserve the graph meta-path architectural information when embedding the network into a low dimensional space; thus, the learned embeddings could be applied to other downstream tasks.

To this end, inspired by the heterogeneous graph attention network (HAN) [23], we propose hierarchical attention schemes, in which we first perform attention at node level to learn the weights of neighbors $N^{\Phi }$ of meta-path $\Phi $, and combine them to obtain the embedding of the semantic-specific node. Then, the difference among meta-paths is computed via semantic-level attention to find the ideal weighted mixture of the semantic-level node embedding for the targeted task.

Owing to node heterogeneity, each type of node has diverse feature spaces. Therefore, for projecting features of each type of node into the same feature space, we compute the node-type transformation matrix $M_{\phi _{i}}$ as expressed in equation (9).

\begin{equation*} h_{i}^{\prime }=M_{\phi _{i}}\cdot h_{i}\tag{9}\end{equation*}

where $h_{i}^{\prime }$ and $h_{i}$ respectively denote the original and projected features of node $i$, and $\phi _{i}$ represents node type. Then, the self-attention mechanism is adopted to capture the weights in between node pairs *(i, j)* with meta-path $\Phi $. So, the relative importance of node $j\in N_{i}^{\Phi }$ for the node, $i$ represents the node-level attention $e_{ij}^{\Phi }$ computed with equation (10) where ${\mathrm {att}}_{\mathrm {node}}$ designates the neural network that accomplishes the node-level attention, which subsequently was normalized with the softmax function to obtain weight coefficient ${\propto }_{ij}^{\Phi }$ as formulated in equation (11),

\begin{align*} e_{ij}^{\Phi }=&{\mathrm {att}}_{\mathrm {node}}\mathrm { (}h_{i}^{\prime },h_{j}^{\prime };\Phi)\tag{10}\\ \propto _{ij}^{\Phi }=&\mathrm {softmax(}e_{ij}^{\Phi }).\tag{11}\end{align*}

Subsequently, the embedding (meta-path) corresponding to node $i$ can be combined by projected features of the neighbors with the respective coefficients as depicted in equation (12),

\begin{equation*} Z_{i}^{\Phi }=\sigma \sum \limits _{j\in N_{i}^{\Phi }} {\propto _{ij}^{\Phi }} \cdot h_{j}^{\prime }\tag{12}\end{equation*}

where $Z_{i}^{\Phi }$ represents the node $i$ learned embedding on the meta-path $\Phi $ via its neighbors. However, such attention only learns on the type of semantic information due to utilizing a single meta-path for calculating attention weight ${\propto }_{ij}^{\Phi }$. Additionally, we observe that the scale-free nature of heterogeneous graphs causes a high variance of graph data. To tackle this problem, we expand node-level attention to multi-head attention to preserve the stability of the training process. We apply the node-level attention $w$ to $K$ times, and the learned embeddings are concatenated to obtain the semantic-specific embedding as formulated in equation (13),

\begin{equation*} Z_{i}^{\Phi }={\vert \vert }_{k=1}^{k}\sigma \left ({\sum \limits _{j\in N_{i}^{\Phi }} {\propto _{ij}^{\Phi }\cdot } h_{j}^{\prime } }\right)\tag{13}\end{equation*}

where $\vert \vert $ denotes concatenation, and $Z=\{Z_{\Phi _{0}},Z_{\Phi _{1}},\cdots, Z_{\Phi _{P}}\}$ represents grouped semantic embedding generated from node-level attention on the $P$ meta-path set $\{\Phi _{0},\Phi _{1},\ldots,\Phi _{P}\}$.

Generally speaking, each node in the graph encompasses several types of semantic information and semantic-specific node embedding that represent a single aspect of node $t$. Also, achieving collective learning of node embedding necessitates the fusion of numerous meta-paths’ semantics. This problem is tackled by applying semantic-level attention that is able to capture the importance of various meta-paths and exploits them for the targeted task. Given the input of $P$ sets generated from node attention, the learned weights of each meta-path {$\beta _{\Phi _{0}},\beta _{\Phi _{1}}, \ldots,\beta _{\Phi _{P}}$} can be expressed with equation (14),

\begin{equation*} \{\beta _{\Phi _{0}},\beta _{\Phi _{1}},\ldots,\beta _{\Phi _{P}}\}= {\mathrm {att}}_{\mathrm {sem}}\{Z_{\Phi _{0}},Z_{\Phi _{1}}\mathrm {,\cdots },Z_{\Phi _{P}}\}.\tag{14}\end{equation*}

We perform a nonlinear transformation on semantic-level embedding and then determine its significance by measuring the resemblance of both transformed embedding and vector $q$ of semantic-level attention. We also calculate the importance of each meta-path as the average of all the semantic-specific node embedding as depicted in equation (15), which is subsequently normalized with equation (16) to obtain $\beta _{\Phi _{i}}$ that represents the influence of the meta-path $\Phi i$ for the molecule graph. The higher value of $\beta _{\Phi _{i}}$ denotes higher importance.

\begin{align*} w_{\Phi _{i}}=&\frac {1}{\left |{ \nu }\right |}\sum \limits _{i\epsilon \nu } q^{T} \cdot \text {tanh}(W\cdot Z_{i}^{\Phi }+b)\tag{15}\\ \beta _{\Phi _{i}}=&\frac {\mathrm {exp(}w_{\Phi _{i}})}{\sum \nolimits _{1}^{P} {\mathrm {exp(}w_{\Phi _{i}})}}\tag{16}\end{align*}

Then we can calculate final embedding $Z$ using the beforementioned semantic-specific embeddings by taking the computed weights as a parameter, as shown in equation (17)

\begin{equation*} Z=\sum \nolimits _{1}^{P} {(\beta _{\Phi _{i}}\cdot Z_{i}^{\Phi })}.\tag{17}\end{equation*}

#### 2) Sequential Learning

In this section, we adopt SMILES to represent the chemical structure of drug compounds in the form of a line notation of atoms and covalent bonds. For instance, the sequence of atoms and covalent bonds is denoted as “CC1=C2C=C(C=CC$\ldots $”. The generated SMILES sequence needs to be encoded to be learned with later deep-learning layers. Several studies adopted one-hot encoding for SMILES tokens [39] but this encoding method ignores the contextual value of the symbols and is therefore unable to expose the operativity of the tokens in the surrounding context. To address this issue, we employed Smi2Vec [41], a method analogous to Word2Vec [42], to encode the tokens in the SMILES sequences. In which fixed-length SMILES symbols detach into a discrete atom, that mapped by finding corresponding embeddings from the pre-trained dictionary or producing a random value if no embedding exists.Finally, atom embedding vectors are aggregated to form the final embedding matrix.

*Bi-Directional ConvLSTM (BConvLSTM):* The embedding representation of drug SMILES $\mathrm {X}^{\mathrm {drug}}$ is now passing into a BConvLSTM layer. The critical shortcoming of the conventional LSTM architecture is that these networks do not consider the spatial association since it utilizes full connections in state-to-state transitions and input-to-state transitions. To tackle this problem, ConvLSTM [24] was introduced, which make use of convolution operations to replace the full connection between various gates. It comprises an input gate $I^{t}$, a forget gate $F^{t}$, and a memory cell $C^{t}$, and an output gate $O^{t}$, while the operation of ConvLSTM can be formulated with equations (18-22),

\begin{align*} I_{t}=&{\sigma (W}_{\mathrm {xi}}\ast X_{t}+W_{hi}\ast H_{t-1}+W_{ci}\ast C_{t-1}+b_{i})\qquad \quad \tag{18}\\ F_{t}=&{\sigma (W}_{\mathrm {xf}}\ast X_{t}+W_{hf}\ast H_{t-1}+W_{\mathrm {cf}}\ast C_{t-1}+b_{f})\tag{19}\\ C_{t}=&{f_{t}\circ C_{t-1}\mathrm {tanh}(W}_{\mathrm {xf}}\ast X_{t}+W_{\mathrm {hc}}\ast C_{t-1}+b_{c})\tag{20}\\ O_{t}=&{\sigma (W}_{\mathrm {xo}}\ast X_{t}+W_{\mathrm {ho}}\ast H_{t-1}+W_{\mathrm {ci}}\circ C_{t-1}+b_{c})\tag{21}\\ H_{t}=&{(O}_{t}\circ \mathrm {tanh}(C_{t}))\tag{22}\end{align*}

where $\ast $ and $\circ $ designate the convolution and Hadamard operation, correspondingly. Here, $X_{t}$ denotes the input tensor (SMILEs embedding), $C_{t}$ and $H_{t}$ represent the memory and the hidden cell tensor, respectively, $W$ and $b$ denote 2D Convolution kernels and bias terms belonging to each cell. For convenience, we eliminate the subscript and superscript from the parameters.

In our architecture, we process the input $X_{t}$ in both forward and backward directions using two ConvLSTMs and then calculate a decision of the current input depending on dual dependencies from both directions. This fully exploits the information in SMILES sequences and so might be effective to improve overall learning performance. Each of the forward and backward ConvLSTMs are regarded as separate ConvLSTM with two sets of parameters for each direction. So, we can calculate the BConvLSTM output with equation (23),

\begin{equation*} Y_{t}=\mathrm {tanh}(W_{y}^{\vec {H}}\ast \vec {H}_{t}+W_{y}^{\stackrel {\leftarrow} {H}}\ast  {\stackrel {\leftarrow} {H}}_{t}+b)\tag{23}\end{equation*}

where $\vec {H}_{t}$, $\mathord {\stackrel {{\lower 3pt\hbox {$\scriptscriptstyle \leftarrow $}}} H} _{t}$ denotes the tensors of hidden state tensors in both the forward and backward units respectively, $b$ represents the bias term, and $Y_{t}\in \mathbb {R}^{F_{t}\times W_{t}\times H_{t}}$ designates the computed spatio-sequential output. Further, tanh implements the hyperbolic tangent for combining states in both directions.

### C. Output Layer

In this part of the network, the final feature representations produced from each channel were concatenated and fed into three FCLs. We build the FCLs with 1024, 768, and 512 nodes for each layer in respective order. After each layer we introduce a regularization dropout layer (0.1) to evade over-fitting by keeping activation for some neurons in the preceding layer. Additionally, we adopt a Rectified Linear Unit (ReLU) as an activation function. Finally, model training attempts to minimize the cost function. Here, we employ mean squared error (MSE) for measuring model loss using equation (24),

\begin{equation*} \mathrm {MSE}=\frac {1}{n} \sum \limits _{i=1}^{n} {(P_{i}-Y_{i})}^{2}\tag{24}\end{equation*}

where $P$ denotes the predicted affinities vector, $Y$ represents the actual outputs, and $n$ is the total number of samples.

## IV. Experiments

Given the set of DT pairs and the corresponding affinity $s$ in a training data, the DeepH-DTA is trained to minimize the objective function presented in equation (24). For a generalized learning purpose, we have arbitrarily divided the dataset into six similar chunks, wherein a single chunk is designated as the self-governing test set. The other chunks of the data are employed to specify the hyper-parameters (as presented in the next section) by means of 5-fold cross validation.

To assure performance robustness, we evaluate the model on leave out the test set and utilize the other five sets of 5-fold cross validation for training the proposed deepH-DTA using the parameters presented in Table 1. Our experiments were performed on Windows 10 (4.2GHz Intel(R) Xeon(R) and Nividia Quadro (4GB)). Implementation details along with the train and test folds (and source codes) of the datasets can be accessed in the link: https://github.com/Hawash-AI/deepH-DTA. The DeepH-DTA takes the protein sequence as a first input. The input molecule is two-folded: a SMILES representation of the molecule and a graph representation of the molecule generated by RDKit. However, the stereochemistry of SMILES (i.e. where some of SMILES representations are not stereospecific). So, in this work, we eliminate stereochemistry in the SMILES input; since the number of relevant cases represent an unimportant percentage of the entire data. Additionally, the graph representation identifies bonds and atoms to account for stereochemistry.

**TABLE 1:** The Hyperparameters of DeepH-DTA

Hyperparameters	Optimal values
No. dense blocks	3
No. conv layer (each block)	3
Filters (each block)	[6, 9, 12]
dropout	0.1
squeeze-and-excitation (threshold)	2
HGAT depth	4
No. BConvLSTM layers	100
Proteins sequence Length	1000
SMILES sequence Length	1000
FCLs	1024; 768; 512
epochs	200
batch size	256
dropout	0.1
optimizer	Adam
learning rate (lr)	0.00001

### A. Model Hyperparameters

Overall implementation of our model conducted using Pytorch library, we initialize bias value with zeros and adopt random weights initialization. Depending on the highest results obtained from several experiments, we discover the optimal hyperparameter setting for our architecture, which is analyzed and discussed in the next section. Table 1 summarizes the optimal hyperparameters of our model.

### B. Datasets

To assess the performance of the proposed approach, we adopt two broadly used benchmark datasets for DTI, namely the DAVIS dataset [43] and KIBA dataset [44]. The Davis dataset encompasses protein samples belonging to the kinase family and their inhibitors along with corresponding dissociation constant $\left ({K_{d} }\right)$ values. In this paper, for numerical stability, we transform the $K_{d}$ values in the DAVIS dataset into log space, $pK_{d}=-\log _{10}\frac {K_{d}}{1\times 10^{9}}$, as proposed by Wang *et al*. [12]. On the other hand, the KIBA dataset integrates various sources of inhibitor bioactivities for optimizing following consistency between $K_{i}$, $K_{d}$, and ${\mathrm {IC}}_{50}$ by applying their statistical information. Table 2 provides a summary of both data sets and adopted splits for our model.

**TABLE 2:** Summary of Experimental Datasets

Data Sets	No. Comps	No. Proteins	No. Inters	Split	No. samples
DAVIS	68	422	30,056	Train	20,037
				Validation	5,009
				Test	5,010
KIBA	2111	229	118,254	Train	78,836
				Validation	19,709
				Test	19,709

Comps= (compounds), Inter=(interactions)

### C. Evaluation Matrices

We use several metrics used for evaluating the performance of our model, which are reliable with those used in previous studies. The computation of these metrics is as follows.

• Concordance Index (CI): used to measure whether the order of estimated binding affinity scores of couples of drugs–the target is identical to the order of true values, and we handle statistical significance using a paired $t$-test with 95% confidence interval (the larger value of CI indicate better model performance). The calculation of CI is in accordance with equation (25),
  \begin{equation*} \mathrm {CI}=\frac {1}{Z}\sum \limits _{\delta _{i}>\delta _{j}} {h(b_{i}-b_{j})}\tag{25}\end{equation*}
  where $b_{i}$, $b_{j}$ represents the prediction score for the higher affinity $\delta _{i}$ and lower affinity $\delta _{j}$, respectively, $Z$ denotes a normalization constant, and the step function $\mathrm {h(x)}$ can be formulated with equation (26)
  \begin{align*} h\left ({x }\right)=\begin{cases} 1,&\mathrm {if}~x>0 \\ 0.5,&\mathrm {if}~x=0 \\ 0,&\mathrm {if}~x < 0. \\ \end{cases}\tag{26}\end{align*}
• Mean Squared Error (MSE): represents the average of differences between predicted and actual output values (the smaller, the better).
• $R$ squared $r_{m}^{2}$: denotes the external prediction performance of the model. Meanwhile, the model is acceptable only when $r_{m}^{2}>0.5$, and $r_{m}^{2}=r ^{2}\times \left ({1-\sqrt {r ^{2}-r_{0}^{2}} }\right)$, where $r ^{2}~\mathrm {and}~r_{0}^{2}$ designate the squared correlation coefficient parameters for the predicted and actual values with and without intercept.
• The area under the precision curve (AUPC): a widely adopted measure for binary classification studies. In an attempt to measure the AUPR for our model, we transformed prediction datasets into binary datasets specifying the threshold for binding affinity for each one. Thus, we select $pK_{d}$ values of 7 and 12.1 as a threshold for the Davis and KIBA datasets, respectively. We choose these values based on because of its proven optimality and wide adoption in previous studies [7], [29], [13].

### D. Results and Comparisons

For demonstrating the competitiveness of our model, we conduct an end-to-end comparison with the cutting-edge approaches (either machine- or deep-learning approaches) adopted for predicting affinity scores, and we conducted the comparative experiments under the same conditions. In Table 3 and Table 4, we provide the average obtained CI, MSE, $r_{m}^{2}$, and AUPC corresponding to each study on the Davis and KIBA datasets, respectively. It can be noted that machine-learning models such as KronRLS and SimBoost show worse performance compared to other deep-learning approaches. This is owing to their dependence on similarity matrices between drugs and targets as well as hand-crafted features. On the other hand, deep-learning techniques that automatically capture feature representation show great performance improvement.

**TABLE 3:** Model Comparison With Cutting Edge Approaches on the DAVIS Dataset

Models	CI (std)$\uparrow $	MSE$\downarrow $	$ {r}_{{m}}^{ {2}}$ (std) $\uparrow $	AUPC (std)$\uparrow $
KronRLS [27]	0.869 (0.001)	0.379	.407 (0.005)	0.661 (0.010)
SimBoost [26]	0.873 (0.002)	0.282	0.644 (0.006)	0.709 (0.008)
String Representation Based Approaches				
DeepDTA [29]	0.878 (0.004)	0.261	0.630 (0.017)	0.714 (0.010)
MT-DTI (wo-FT) [32]	0.875 (0.003)	0.268	0.633 (0.013)	0.700 (0.011)
MT-DTI [32]	0.887 (0.001)	0.245	0.665 (0.014)	0.730 (0.014)
DeepCPI [28]	0.867	0.293	0.607	0.705
WideDTA [7]	0.886 (0.003)	0.262	0.633 (0.011)	0.711 (0.012)
GANsDTA [13]	0.881 (0.005)	0.276	0.653 (0.015)	0.653 (0.017)
Attention-DTA [14]	0.887 (0.005)	0.245	0.657 (0.024)	0.746 (0.024)
Graph Representation Based Approaches				
GAT [11]	0.892 (0.003)	0.232	0.662 (0.010)	0.728 (0.016)
GIN [11]	0.893 (0.003)	0.229	0.649 (0.013)	0.720 (0.016)
GIN [12]	0.899 (0.003)	0.220	0.623 (0.011)	0.726 (0.015)
DeepGS [30]	0.880 (0.005)	0.252	0.686 (0.012)	0.763 (0.012)
DeepH-DTA*	0.924 (0.001)	0.195	0.725 (0.009)	0.801 (0.010)

The * denote the proposed architecture

**TABLE 4:** Model Comparison With Cutting Edge Approaches on the KIBA Dataset

Models	CI (std)$\uparrow $	MSE$\downarrow $	${r}_{ {m}}^{ {2}}\mathrm {(std)} {\uparrow }$	AUPC (std)$\uparrow $
KronRLS [27]	0.782 (0.001)	0.411	0.342(0.001)	0.635 (0.004)
SimBoost [26]	0.836 (0.001)	0.222	0.629(0.007)	0.760 (0.003)
String Representation Based Approaches				
DeepDTA [29]	0.863 (0.002)	0.194	0.673(0.009)	0.788 (0.004)
MT-DTI (wo-FT) [32]	0.844 (0.001)	0.220	0.584 (0.002)	0.789 (0.004)
MT-DTI [32]	0.882 (0.001)	0.220	0.584 (0.003)	0.789 (0.006)
DeepCPI [28]	0.852 (0.002)	0.211	0.657 (0.004)	0.782 (0.005)
WideDTA [7]	0.875 (0.001)	0.179	0.675 (0.005)	0.788 (0.008)
GANsDTA [13]	0.866 (0.001)	0.224	0.775 (0.008)	0.753 (0.007)				
Attention-DTA [14]	0.882 (0.004)	0.162	0.735 (0.003)	0.829 (0.005)
Graph Representation Based Approaches				
GAT [11]	0.889 (0.001)	0.139	0.671 (0.005)	0.781 (0.006)
GIN [11]	0.891 (0.004)	0.139	0.684 (0.004)	0.801 (0.005)
GIN [12]	0.901 (0.002)	0.129	0.680 (0.003)	0.799 (0.004)
DeepGS [30]	0.860 (0.003)	0.193	0.684 (0.002)	0.801 (0.005)
DeepH-DTA*	0.927 (0.003)	0.111	0.799 (0.004)	0.861 (0.002)

The * denote the proposed architecture

First, this paper considers a few recent textual representation approaches such as: DeepDTA [29], MT-DTI [32], Deep-CPI [28], WideDTA [7], GANsDTA [13], and Attention-DTA [14]. Among these approaches, Attention-DTA and MT-DTI yielded best results with CI of 0.887, MSE of 0.245 on the Davis dataset; also, on the KIBA dataset, they both achieved CI of 0.882 and MSE of 0.220 and 0.162 respectively. This explains the effectiveness of the attention convolutional operation in learning sequential drug and target information in the case of Attention-DTA [14]; and also, the efficiency of the pre-trained BERT representation presented in MT-DTI.

Second, graph network approaches [11], [12], [16] can effectively capture topological relationships of drug molecules, which enable further performance improvement. Amongst them, the GIN [12] shows a higher CI value of 0.899 and lower MSE of 0.222 on the Davis dataset; and 0.901 of CI and 0.129 of MSE on the KIBA dataset. Meanwhile, DeepGS [30] yield the least performance with CI of 0.880 and 0.860 and MSE of 0.252 and 0.193, respectively, on the Davis and KIBA datasets.

It can be observed that the proposed DeepH-DTA has a robust performance on both datasets, achieving 0.924 (0.025 improvement), 0.195 (reduced by 0.025), 0.725 (0,039 improvement), and 0.801(0.038 improvement) for CI, MSE, $r_{m}^{2}$, and AUPC, respectively, for the Davis dataset. For the KIBA dataset, we achieved 0.927 for CI (0.026 improvement), 0.111 for MSE (reduced by 0.018), 0.799 for $r_{m}^{2}$ (0.024 improvement), and 0.861 for AUPC (0.032 improvement).

For the Davis dataset, the proposed DeepH-DTA outperforms the traditional ML techniques [26], [27] by 5% of CI and with statistical significance ($p$-value of 0.00009 for both). Further, DeepH-DTA outperforms WideDTA [7] by 4% of CI score with statistical significance ($p$-value, 0.002). Also, it outperforms the best graph-based approach GIN [12] by 2.5% of CI with statistical significance ($p$-value, 0.01). On the other hand, for the KIBA dataset, it outperforms both of these techniques by >9% with statistical significance ($p$-value around 0.0005 for both [26] and [27]). For the string-based approach, DeepH-DTA outperforms WideDTA [7] by 5.2% of CI score with statistical significance ($p$-value, 0.004). Further, it outperforms the best graph-based approach GIN [12] by 2.6% of CI with statistical significance ($p$-value, 0.009).

In order to further verify model performance, we note that DeepH-DTA attains 2.5% of MSE lower than the lowest existing MSE in GIN [12] with statistical significance ($p$-value, 0.007) on the Davis dataset. Also, it achieves 1.8% of MSE lower than the lowest current MSE in GIN [12] with statistical significance ($p$-value, 0.01) on KIBA dataset. This indicates the superiority of our proposed approached compared to the most recent studies for predicting DTA. Accordingly, we observe that our model outperforms existing deep-learning methods on four measures, which can be explained due to several factors:

1) In comparison with DeepCPI, our model cooperatively exploits the drugs’ topological structures along with following characteristics of chemical context, which in turn significantly improves the performance.
2) Compared with both Deep-DTA architectures, we adopted the HGAT architecture to learn the structural information of the drug, and employed innovative embedding methods to obtain extra contextual information for both drugs and protein sequences.
3) Compared with graph-based approaches [11], [12], [30], the proposed dense network with squeeze-and-excitation operation models protein sequence information more effectively compared to traditional CNN. Further, utilizing HGAT allows better exploitation of semantic information in meta-path data. Also, Bi-ConvLSTM allows for better exploitation of spatio-sequential representation from SMILEs sequences.

**FIGURE 2.**
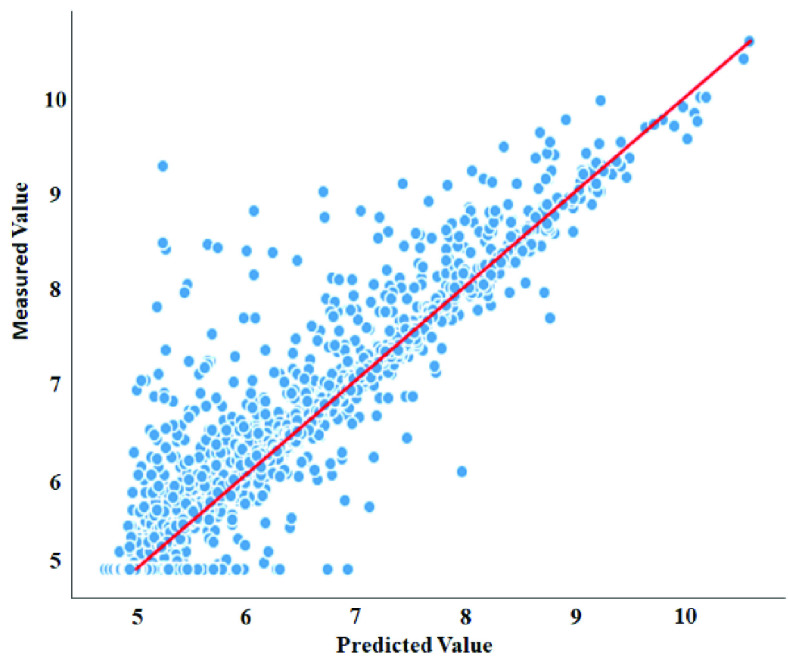
Predictions from our model against measured (real) binding affinity values for Davis dataset ($pK_{d}$).

**FIGURE 3.**
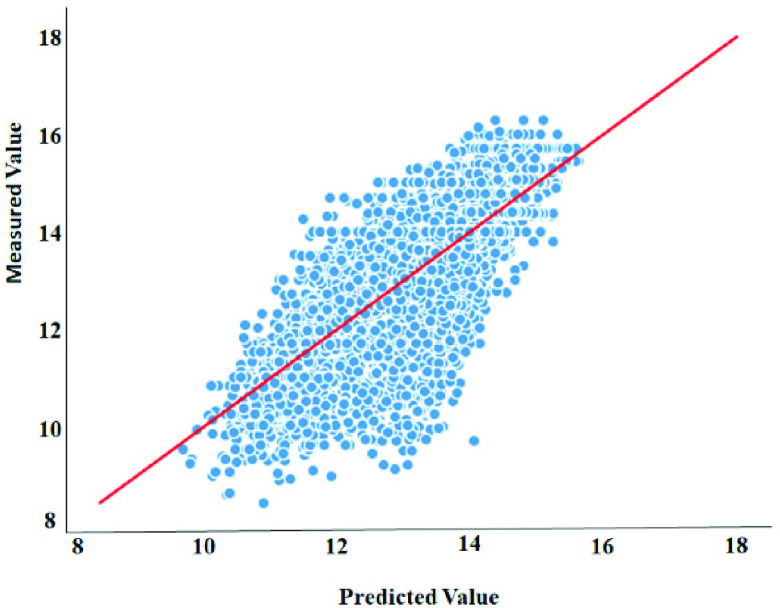
Predictions from our model against measured (real) binding affinity values for the KIBA dataset (KIBA score).

Generally, the obtained results and comparisons demonstrate that our model achieves competitive performance outperforms against these baselines methods in all metrics.Moreover, Fig. 2 and Fig. 3 present the scatter plots of the proposed model predicted affinity score against the actual measured value on the DAVIS and KIBA datasets. The model achieves better performance when the estimated affinity scores are close to the original scores, and hence the instances should appear close to the red line. With regard to the DAVIS dataset, it can be observed that the greater number of the ${pK}_{d}$ scores are found in the range of [5], [6] along the x-axis, principally because the ${pK}_{d}$ score of 5 establishes more than half of the dataset. Additionally, there is a crowded area of KIBA scores lying in the range [11], [14] along the x-axis, which shows similar behavior to the Davis dataset. Principally, for both datasets, the data instances are close to the red regression line which, in turn, demonstrates that the proposed architecture has a competitive prediction performance.

#### 1) Impact of Dense & SE Block

**FIGURE 4.**
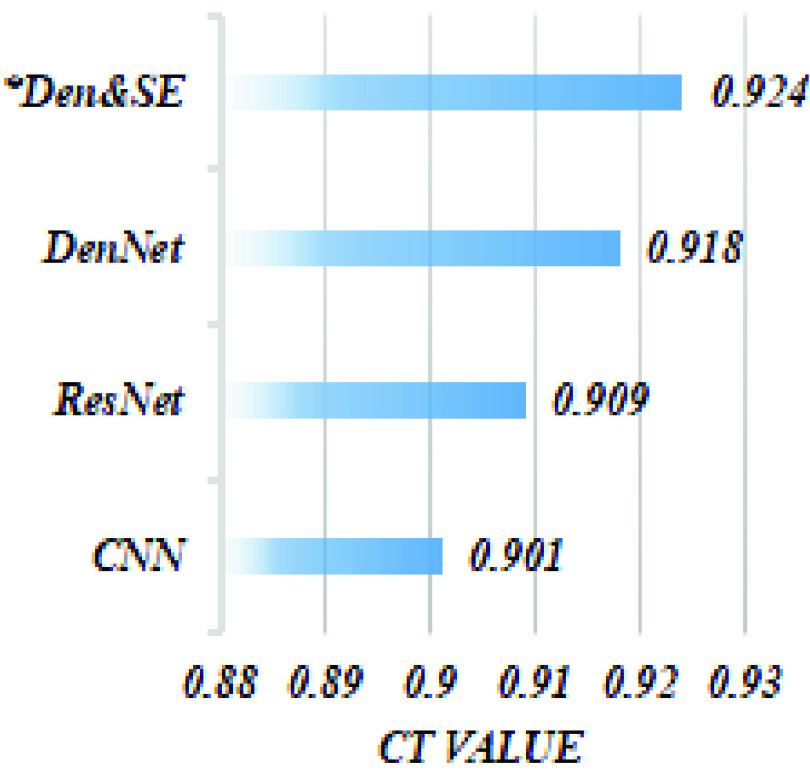
The CI value attained by implementing DeepH-DTA using different CNN implementations on the Davis dataset.

In Fig. 4 we show the result of model implementation using a different CNN architecture—namely: traditional CNN (CNN), Residual CNN (ResNet), and Dense CNN (DenseNet)—and it can be observed that employing squeezed-excited operation after each dense block improves model performance compared to other architectures due to exploiting multi-channel dependency and hence capturing interrelationships of protein features. It could be noted that the Dense Net architecture attains 0.017 higher CI than traditional CNN and 0.009 higher than Residual implementation. This explains the effectiveness of collective learning of dense networks in learning protein sequences. Additionally, the proposed Dense net with SE block attains a further 0.016 improvement.

#### 2) Impact of HGAT

**FIGURE 5.**
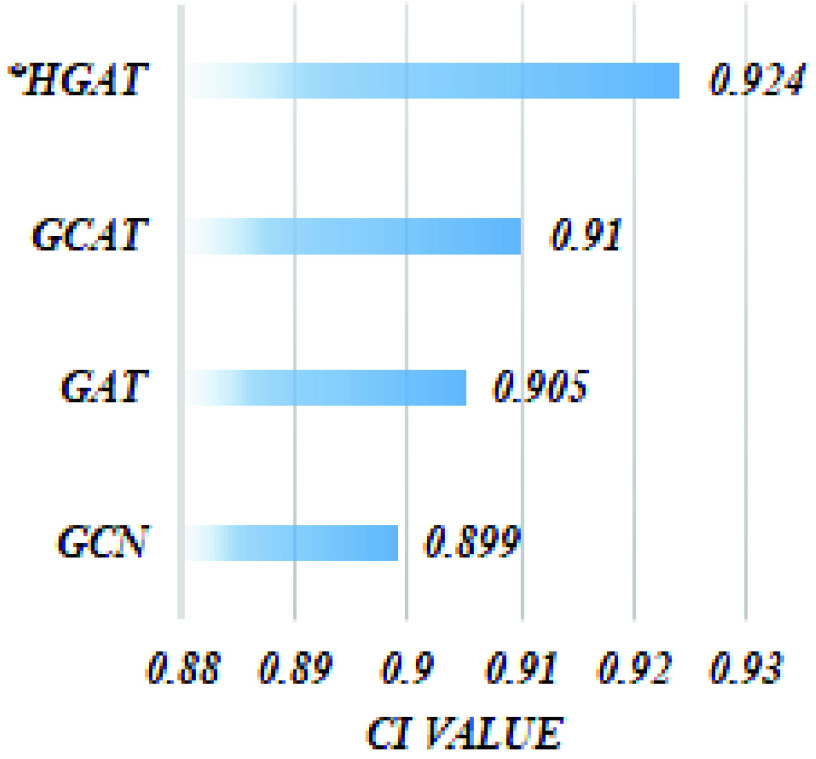
The CI value attained by implementing DeepH-DTA using different types of GN on the Davis dataset.

In order to demonstrate the efficiency of the proposed graph neural network (GNN), we implemented different versions of our architecture utilizing various types of a graphical network, particularly GCN [60], GAT [61], and hybrid architecture (GCAT). The GCN consist of a novel variant of CNN that effectively operates on graph data, whereas the GAT performs similar operations by applying self-attentional layers to attend to the features of the node’s neighborhoods. The corresponding results are shown in Fig. 5 which shows that the proposed HGAT adopted in our model significantly improves model performance by 14% owing to applying semantic attention mechanism on meta-path.

#### 3) Impact of ConvLSTM

**FIGURE 6.**
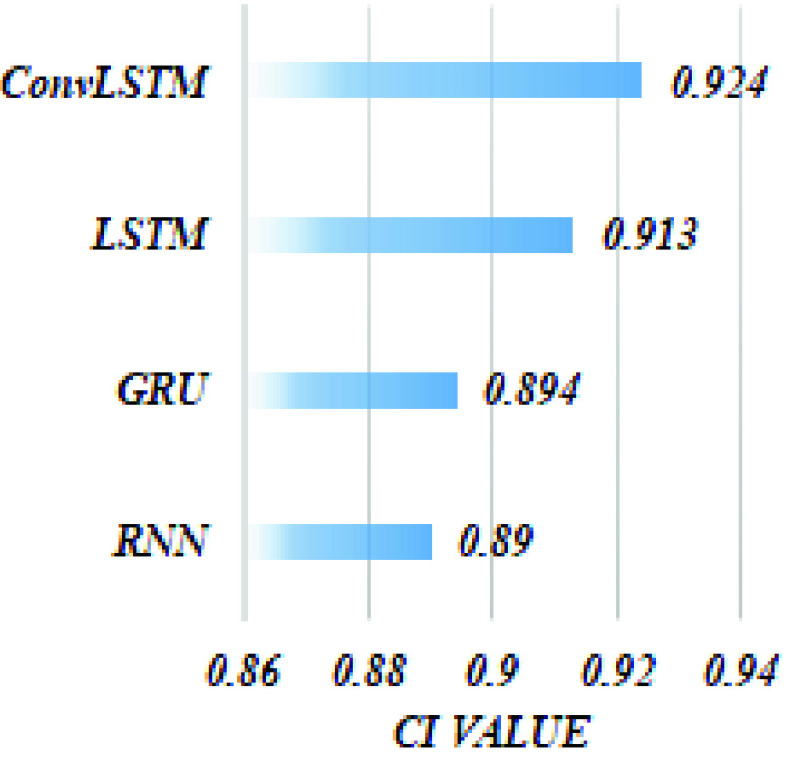
The CI value attained by implementing DeepH-DTA using different types of RNN on the Davis dataset.

**FIGURE 7.**
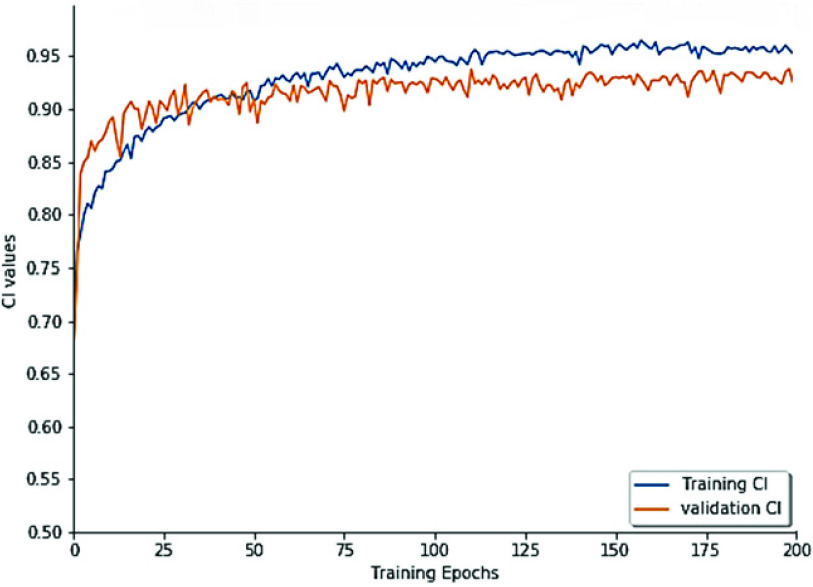
Model’s CI training progress on Davis dataset.

**FIGURE 8.**
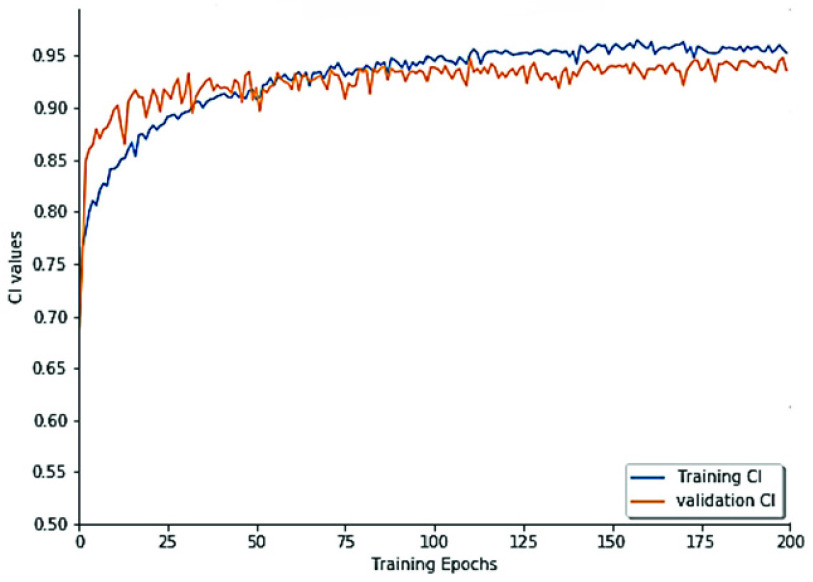
Model’s CI training progress on the KIBA dataset.

**FIGURE 9.**
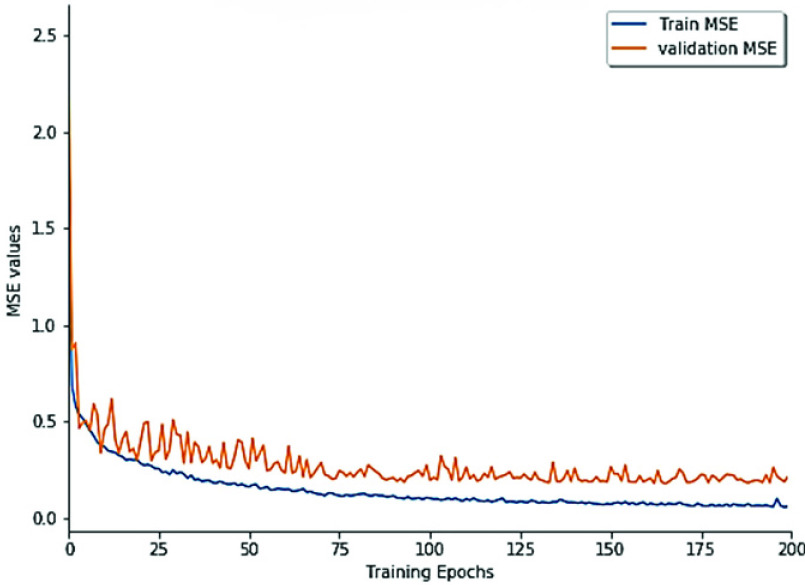
Model’s MSE training progress on the Davis dataset.

**FIGURE 10.**
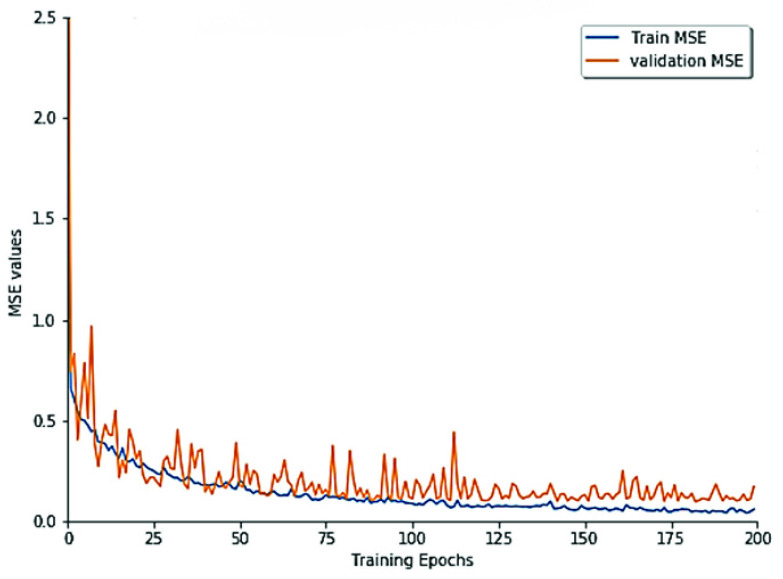
Model’s MSE training progress on the KIBA dataset.

Further, in an attempt to verify our hypothesis about the effectiveness of ConvLSTM in capturing spatio-sequential information from input SMILES. We evaluate the performance of the proposed DeepH-DTA on DAVIS dataset using different versions of RNNs as presented in Fig. 6. It could be noted that simple RNN attains the lowest CI value with 0.886, and 0.008 improvements are achieved when using GRU. Also, an extra improvement with 2.3% could be observed when using LSTM while attaining the maximum CI value when ConvLSTM is employed to implement DeepH-DTA with 0.924 of CI outperforming the LSTM performance by 1.1%. This experiment demonstrates the effectiveness of using ConvLSTM for modeling the SMILES string input.Furthermore, in Fig. 7 and Fig. 8, we present model training progress in terms of CI values corresponding to the Davis and KIBA dataset correspondingly. On both datasets, we observe rapid validation convergence after 100 epochs. It could be noted that the model validation CI is higher than training CI at first 25 epochs on the Davis dataset, and show similar behavior with the early 50 epochs on the KIBA dataset. The training CI values maintain a higher value than validation CI. Fig. 9 and Fig. 10 display the training progress in terms of MSE loss on the Davis and KIBA datasets. On the Davis dataset, we observe early convergence after 120 epochs; meanwhile, start confluence after 125 epochs of training on the KIBA dataset. Our model always has training MSE lower that validation MSE on both datasets. However, it shows the opposite behavior on the first 35 epochs of training on KIBA dataset. Finally, we observe that the progress of validation MSE on the Davis dataset is more stable than the KIBA dataset.

**FIGURE 11.**
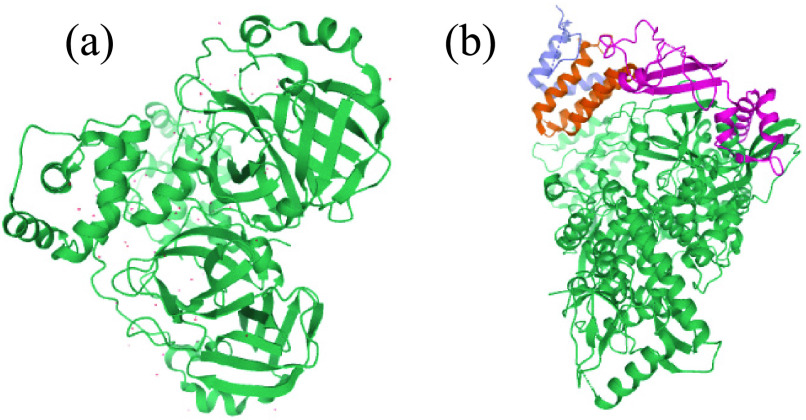
(a) 3D View of the PDB ID: 6WQF. Fig. 11. (b) 3D View of the NCBI: YP_009725307.1.

### E. Computational Complexity

The most advantageous characteristic of deep learning approaches is that they could be executed and trained using the Graphics Processing Unit (GPU). Concerning time complexity, GPU-based deep-learning approaches show a significant reduction in time complexity compared to the case when running on a traditional CPU. Gawehn *et al*. [62] discussed and introduced several strategies for employing GPU to accelerate drug discovery systems. For further verification of the effectiveness of the proposed DeepH-DTA, the computational complexity needs to be addressed. In this regard, we compare the execution time (time in seconds/epoch) of the DeepH-DTA against the before mentioned graph-representation-based approaches, as presented in Table 5. It can be noted that the GIN based approaches [11], [12] consume the least execution time on both datasets. Further, the proposed DeepH-DTA consumes comparable execution time to the GAT [11], and the DeepGS [30]. This could be explained due to the time taken to calculate attention at meta-paths and the time consumed for spatio-sequential learning using ConvLSTM. Compared to the attained performance improvements, this slight increase in execution time indicates the effectiveness and the ability to integrate the DeepH-DTA in real-life scenarios.

**TABLE 5:** Comparison Between Average Execution Time (Second / Epoch)

Dataset / Models	GAT [11]	GIN [11]	GIN [12]	DeepGS [30]	DeepH-DTA*
DAVIS	391s	198s	209s	404s	426s
KIBA	1431s	706s	692s	1530s	1556s

Furthermore, DeepH-DTA does not necessitate matrix factorization or resemblance matrices, hence it offers further scalability compared to the SimBoost and the KronRLS. Given $m$ represents the number of protein sequences and $n$ represents the number of compounds, meanwhile SimBoost and KronRLS and require the resemblance matrices, which have space and time complexity of $O(n^{2}+m^{2})$. SimBoost includes matrix factorization and thereby represents additional expense. On the other hand, in every epoch of DeepH-DTA training procedures, the time complexity only be contingent on the number of DT pairs in the training set which, in the highest situation, is $O\mathrm {(max(}n,m\mathrm {))}$, and $O(nm)$ in the worst situations. There is no clearly formulated interrelationship between the count of epochs while waiting for convergence and $n~\mathrm {or}~m$, thus the count of epochs could not be investigated hypothetically. Nevertheless, it is notable that, in practice, such count is probably sub-linear in $n$ and $m$ or even autonomous from $n$ and $m$, so the epochs count can be statistically set to a slight constant if we aim to realize comparatively primitive results, whereas the SimBoost and KronRLS firmly necessitate a minimum of $O(n^{2}+m^{2})$ time to attain any results.

## V. SARS-COV-2 Drug Repurposing

### A. Modeling Drug and SARS-COV-2 Interactions

In this section, we apply our proposed model for predicting binding affinity scores for commercially existing drugs, and SARS-CoV-2 proteins in order to identify the best inhibitors that can suppress virus spread and provide scientists with a start point for developing new vaccines. For this purpose, we collected several amino acid sequences from the Protein Data Bank (PDB) database and the National Center for Biotechnology Information (NCBI), as listed in Table 6.

**TABLE 6:** Model Comparison With Cutting Edge Approach on KIBA Dataset

Proteins	Identifier
3C-like proteinase	PDB ID: 6WQF
RNA-dependent RNA polymerase (RdRp)	NCBI: YP_009725307.1

Then the proposed DeepH-DTA is trained on two public databases that are manually combined: namely, the journal curated Binding DB [45] and the Drug Bank database (interactions: 26167; drugs: 7591; target: 4187) [5] with three types of efficiency scores as in the KIBA dataset. The DeepH-DTA is trained for 75 epochs using the same hyperparameters presented in Table 1 and under the same experimental conditions discussed in the previous section. To attain rapid convergence, the DeepH-DTA parameters were initialized by transferring the learned parameters from the KIBA dataset. Further, we average the consistence-score procedure [46] to integrate these scores and keep their Pearson correlation score above 0.9. Since the aggregated data involves extensive heterogeneity of molecules and proteins, the proposed DeepH-DTA has inherent superiority for modeling the interactions between antiviral medications and protein sequences of SARS-COV-2. After that, the DeepH-DTA predictions were filtered out for FDA-approved drugs with the highest binding to target viral proteins. Moreover, we included Remdesivir and Ivermectin because therapeutic potential to COVID-19 has been proposed recently in [48], [49], and we also included drugs from clinical trials.

### B. Findings and Discussions

We exploit the advantage of heterogonies for modeling DTI to predict affinity scores of 3,001 FDA-approved drugs against 3C_pro_, RdRp, helicase, 30-to-50 exonuclease, endoRNAse, and 2OMT of SARS-CoV-2. For a better understanding of these genes, please refer to section 1(B).

**FIGURE 12.**
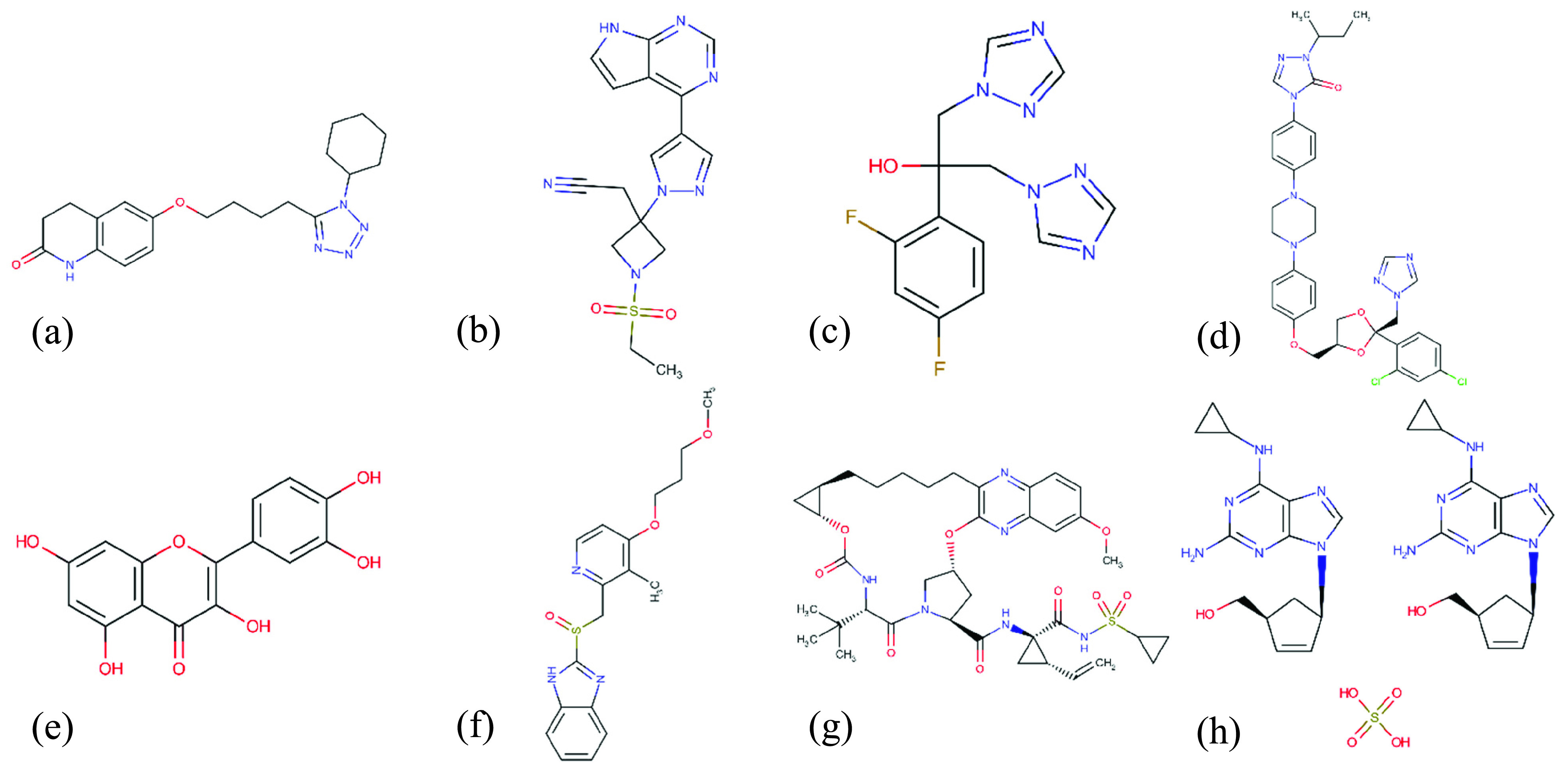
Structural formulas of candidate drugs against SARS-CoV-2 3C-like proteinase (a) Cilostazol, (b) Baricitinib, (c) Fluconazole, (d) Itraconazole, (e) Quercetin, (f) Rabeprazole, (g) Grazoprevir, (h) Abacavir (sulfate).

Table 7 and Table 8 present the top inhibitor list for SARS-CoV-2 main-protease and RdRp proteins, respectively. Both tables provide the commercial drug name, corresponding SMILES format, models’ predicted affinity scores ($K_{d}$ in nM), and the clinical evidence for this prediction if exist (clinical approval means the research study that proves the effectiveness of the certain drug against COVID-19). In Table 7, we observe that SARS-CoV-2 main-protease was estimated to bind with Cilostazol ($K_{d}$: 53.13 nM), Baricitinib ($K_{d}$: 59.27 nM), Fluconazole ($K_{d}$: 64.34 nM), Itraconazole ($K_{d}$: 70.35), Quercetin ($K_{d}$: 79.24 nM), Rabeprazole ($K_{d}$: 85.26 nM), Grazoprevir ($K_{d}$: 79.24 nM) and other drugs with a prediction affinity of over 100 nM. Additionally, we present Structural graphical formulas of some of suggested drugs in Fig. 12.

**TABLE 7:** DTI Prediction Results of FDA Approved Antiviral Drugs 3C-Like Proteinase of SARS-CoV-2

Smal molecule	SMILES forma	Predicted $\text{K}_{\text {d}}$ in nM	Clinical approved	DOSE
Cilostazol	O=C1CCC2=C(N1)C=CC(OCCCCC1=NN=NN1C1CCCCC1)=C2	53.1	–	100 mg twice/daily
Baricitinib	CCS(=O)(=O)N1CC(C1)(CC#N)N2C=C(C=N2)C3=C4C=CNC4=NC=N3	59.2	Clinical trials, [49], [50]	2 mg once / daily
Fluconazole	OC(CN1C=NC=N1)(CN1C=NC=N1)C1=C(F)C=C(F)C=C1	64.34	–	100 - 400 mg/daily
Itraconazole	CCC(C)N1N=CN(C1=O)C1=CC=C(C=C1)N1CCN(CC1)C1=CC=C(OC[C@H]2CO[C@@](CN3C=NC=N3)(O2)C2=CC=C(Cl)C=C2Cl)C=C1	70.3	–	100 - 400 mg/daily
Quercetin	OC1=CC2=C(C(O)=C1)C(=O)C(O)=C(O2)C1=CC=C(O)C(O)=C1	79.24	Clinical trial, [57]	500 −1000 mg/daily
Rabeprazole	[Na+].COCCCOC1=CC=NC(CS(=O)C2=NC3=CC=CC=C3[N-]2)=C1C	85.2	Clinical trial	120 mg/daily
Grazoprevir	COC1=CC2=NC3=C(CCCCC[C@@H]4C[C@H]4OC(=O)N[C@H](C(=O)N4C[C@@H](C[C@H]4C(=O)N[C@@]4(C[C@H]4C=C)C(=O)NS(=O)(=O)C4CC4)O3)C(C)(C)C)N=C2C=C1	98.47	[51]	100 mg/day
Abacavir (sulfate)	OS(O)(=O)=O.NC1=NC2=C(N=CN2[C@@H]2C[C@H](CO)C=C2)C(NC2CC2)=N1.NC1=NC2=C(N=CN2[C@@H]2C[C@H](CO)C=C2)C(NC2CC2)=N1	107.62	–	300 mg twice /day
Bortezomib	CC(C)C[C@H](NC(=O)[C@H](CC1=CC=CC=C1)NC(=O)C1=CN=CC=N1)B(O)O	117.23	[52]	1.3 mg/12 hour
Metoprolol tartrate	[H][C@](O)(C(O)=O)[C@@]([H])(O)C(O)=O.COCCC1=CC=C(OCC(O)CNC(C)C)C=C1.COCCC1=CC=C(OCC(O)CNC(C)C)C=C1	129.23	–	160 mg/day
Rifabutin	CO[C@H]1\C=C\O[C@@]2(C)OC3=C(C)C(O)=C4C(O)=C(NC(=O)\C(C)=C/C=C/[C@H](C)[C@H](O)[C@@H](C)[C@@H](O)[C@@H](C)[C@H](OC(C)=O)[C@@H]1C)C1=C(N=C5C=C(C)C=CN15)C4=C3C2=O	145.19	–	60 mg/day
Ritonavir	CC(C)C1=NC(=CS1)CN(C)C(=O)NC(C(C)C)C(=O)NC(CC2=CC=CC=C2)CC(C(CC3=CC=CC=C3)NC(=O)OCC4=CN=CS4)O	163.36	[54]–[56]	50 to 100 mg twice/day
Tetraethylene glycol	C(COCCOCCOCCO)O	171.14	Clinical trials	2000 mg/day
Adenosine monophosphate	C1=NC(=C2C(=N1)N(C=N2)C3C(C(C(O3)COP(=O)(O)O)O)O)N	178.32	–	500 mg/day
Lopinavir	CC(C)[C@H](N1CCCNC1=O)C(=O)N[C@H](C[C@H](O)[C@H](CC1=CC=CC=C1)NC(=O)COC1=C(C)C=CC=C1C)CC1=CC=CC=C1	183.14	[55]–[56]	800 mg/day
Chloroquine phosphate	OP(O)(O)=O.CCN(CC)CCCC(C)NC1=CC=NC2=CC(Cl)=CC=C12	189.35	[47]	500 mg / week
Atazanavir	COC(=O)N[C@H](C(=O)N[C@@H](CC1=CC=CC=C1)[C@@H](O)CN(CC1=CC=C(C=C1)C1=CC=CC=N1)NC(=O)[C@@H](NC(=O)OC)C(C)(C)C)C(C)(C)C	195.57	–	400 mg / day
Remdesivir	CCC(CC)COC(=O)[C@H](C)N[P@](=O)(OC[C@H]1O[C@](C#N)([C@H](O)[C@@H]1O)C1=CC=C2N1N=CN=C2N)OC1=CC=CC=C1	201.13	[51]	200 mg / day

**TABLE 8:** DTI Prediction Results of FDA Approved Antiviral Drugs RdRp of SARS-CoV-2

Smal Molecule	SMILES forma	Predicted $\text{K}_{\text {d}}$ in nM	Clinical approved	Dose
Sirolimu	CC1CCC2CC(C(=CC=CC=CC(CC(C(=O)C(C(C(=CC(C(=O)CC(OC(=O)C3CCCCN3C(=O)C(=O)C1(O2)O)C(C)CC4CCC(C(C4)OC)O)C)C)O)OC)C)C)C)OC	8.13	Clincal trials, [19]	2 mg / day
Ivermectin	CO[C@H]1C[C@@H](O[C@@H](C)[C@@H]1O)O[C@H]1[C@H](C)O[C@H](C[C@@H]1OC)O[C@H]1[C@@H](C)\C=C\C=C2/CO[C@@H]3[C@H](O)C(C)=C[C@@H](C(=O)O[C@H]4C[C@@H](C\C=C1/C)O[C@@]1(CC[C@H](C)[C@@H](C(C)C)O1)C4)[C@]23O.CC[C@@H](C)[C@H]1O[C@@]2(CC[C@@H]1C)O[C@@H]1C\C=C(C)\[C@@H](O[C@@H]3O[C@@H](C)[C@H](O[C@@H]4O[C@@H](C)[C@H](O)[C@@H](OC)C4)[C@@H](OC)C3)[C@@H](C)\C=C\C=C3/CO[C@@H]4[C@H](O)C(C)=C[C@@H](C(=O)O[C@@H](C1)C2)[C@]34O	9.94	[48]	15 mg/day
Methylprednisolone	[H][C@@]12CC[C@](O)(C(=O)CO)[C@@]1(C)C[C@H](O)[C@@]1([H])[C@@]2([H])C[C@H](C)C2=CC(=O)C=C[C@]12C	11.26	Clincal trials	60 mg/day
Abacavir (sulfate)	OS(O)(=O)=O.NC1=NC2=C(N=CN2[C@@H]2C[C@H](CO)C=C2)C(NC2CC2)=N1.NC1=NC2=C(N=CN2[C@@H]2C[C@H](CO)C=C2)C(NC2CC2)=N1	16.92	–	300 mg twice /day
Rifaximi	CO[C@H]1\C=C\O[C@@]2(C)OC3=C(C2=O)C2=C(C(O)=C3C)C(=O)C(NC(=O)\C(C)=C/C=C/[C@H](C)[C@H](O)[C@@H](C)[C@@H](O)[C@@H](C)[C@H](OC(C)=O)[C@@H]1C)=C1NC3(CCN(CC3)CC(C)C)N=C21	20.03	–	550 mg three / day
Ritonavi	CC(C)[C@H](NC(=O)N(C)CC1=CSC(=N1)C(C)C)C(=O)N[C@H](C[C@H](O)[C@H](CC1=CC=CC=C1)NC(=O)OCC1=CN=CS1)CC1=CC=CC=C1	23.26	[55]–[56]	50 to 100 mg twice/day
Metoprolol Tartrate	[H][C@](O)(C(O)=O)[C@@]([H])(O)C(O)=O.COCCC1=CC=C(OCC(O)CNC(C)C)C=C1.COCCC1=CC=C(OCC(O)CNC(C)C)C=C1	28.06	–	160 mg/day
Digoxi	[H][C@]12CC[C@]3([H])[C@]([H])(C[C@@H](O)[C@]4(C)[C@H](CC[C@]34O)C3=CC(=O)OC3)[C@@]1(C)CC[C@@H](C2)O[C@H]1C[C@H](O)[C@H](O[C@H]2C[C@H](O)[C@H](O[C@H]3C[C@H](O)[C@H](O)[C@@H](C)O3)[C@@H](C)O2)[C@@H](C)O1	32.01	–	0.1 mg /day
Atazanavir	COC(=O)N[C@H](C(=O)N[C@@H](CC1=CC=CC=C1)[C@@H](O)CN(CC1=CC=C(C=C1)C1=CC=CC=N1)NC(=O)[C@@H](NC(=O)OC)C(C)(C)C)C(C)(C)C	35.67	–	200 mg / day
Ciclesonid	CC(C)C(=O)OCC(=O)C12C(CC3C1(CC(C4C3CCC5=CC(=O)C=CC45C)O)C)OC(O2)C6CCCCC6	36.27	Clinical trials	1.6 mg /day
Dexamethason	[H][C@@]12C[C@@H](C)[C@](O)(C(=O)CO)[C@@]1(C)C[C@H](O)[C@@]1(F)[C@@]2([H])CCC2=CC(=O)C=C[C@]12C	38.02	–	47.59 mg/day
N-Acetyl-beta- D-glucosamine	CC(=O)NC1C(C(C(OC1O)CO)O)O	39.89	Clinical trials	4170 mg/day
Daclatasvir	COC(=O)N[C@@H](C(C)C)C(=O)N1CCC[C@H]1C1=NC=C(N1)C1=CC=C(C=C1)C1=CC=C(C=C1)C1=CN=C(N1)[C@@H]1CCCN1C(=O)[C@@H](NC(=O)OC)C(C)C	42.23	Clinical trials. [50]	60 mg/ day
Rifabutin	CO[C@H]1\C=C\O[C@@]2(C)OC3=C(C)C(O)=C4C(O)=C(NC(=O)\C(C)=C/C=C/[C@H](C)[C@H](O)[C@@H](C)[C@@H](O)[C@@H](C)[C@H](OC(C)=O)[C@@H]1C)C1=C(N=C5C=C(C)C=CN15)C4=C3C2=O	49.12	–	60 mg/day
Remdesivir	CCC(CC)COC(=O)[C@H](C)N[P@](=O)(OC[C@H]1O[C@](C#N)([C@H](O)[C@@H]1O)C1=CC=C2N1N=CN=C2N)OC1=CC=CC=C1	51.13	[51]	400 mg / day
Lopinavir	CC(C)[C@H](N1CCCNC1=O)C(=O)N[C@H](C[C@H](O)[C@H](CC1=CC=CC=C1)NC(=O)COC1=C(C)C=CC=C1C)CC1=CC=CC=C1	53.04	[55]–[56]	800 mg/day
Chloroquine	CCN(CC)CCCC(C)NC1=C2C=CC(=CC2=NC=C1)Cl	58.21	[58]	500 mg / week
Leflunomide	CC1=C(C=NO1)C(=O)NC1=CC=C(C=C1)C(F)(F)F	62.75	Clinical trials. [50], [59]	–
Hydroxychloroquine	CCN(CCCC(C)NC1=C2C=CC(=CC2=NC=C1)Cl)CCO	69.21	[58]	400 mg/day

**FIGURE 13.**
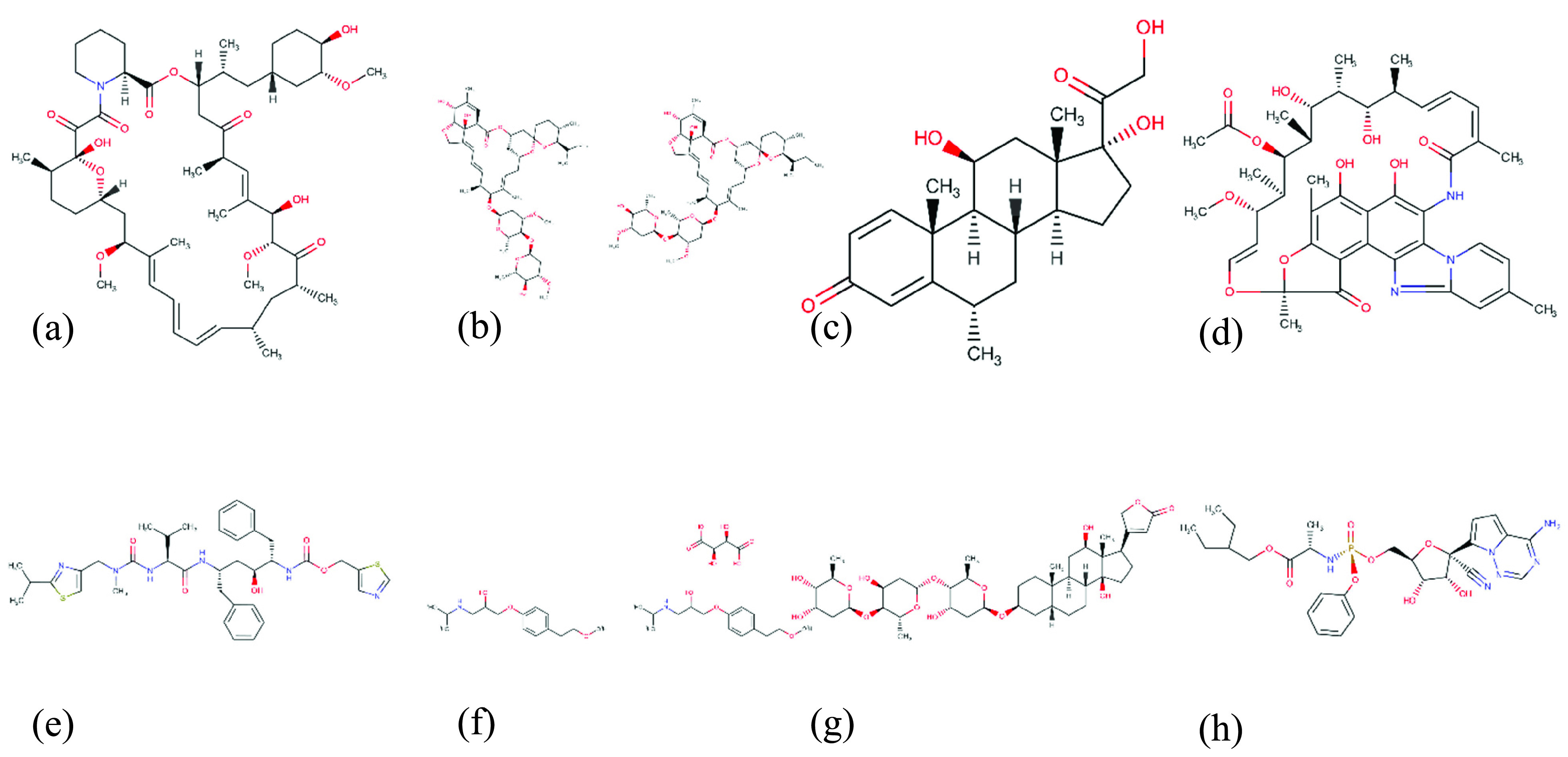
Structural formulas of candidate drugs against SARS-CoV-2 RdRp (a) Sirolimu, (b) Ivermectin,(c) Methylprednisolone, (d) Rifaximin, (e) Ritonavi, (f) Metoprolol Tartrate, (g) Digoxin, (h) Remdesivir.

On the other hand, in Table 8, we introduce the top estimated affinities with RdRp; it can be observed that RdRp of SARS-COV-2 bind with Sirolimus ($K_{d}$: 8.13 nM), Ivermectin ($K_{d}$: 9.94 nM), Methylprednisolone ($K_{d}$: 11.26 nM), Abacavir sulfate ($K_{d}$: 16.92 nM), Rifaximin ($K_{d}$: 20.03nM), Ritonavir ($K_{d}$: 23.26 nM), and Metoprololtartrate ($K_{d}$: 28.06 nM) and some other drug candidates. Also, we present Structural graphical formulas of some suggested drugs in Fig. 13. From the results obtained on both tables with the lowest $K_{d}$ the value represents the drugs with the highest binding affinity against SARS-CoV-2, which can help clinical researchers to investigate these drugs or use them as a starting point to develop a new vaccine.

Several studies used DTI prediction as a tool for drug repurposing to discover novel utilization of current drugs. Accordingly, we make use of our proposed approach to enable controlling the explosion of SARS-CoV-2. Recently, numerous studies have recognized encouraging drug nominees that can assist in inhibiting various aspects of SARS-CoV-2. For example, Baricitinib and Methotrexate revealed inhibitory impacts against SARS-CoV-2 [49], [50]. Also, Grazoprevir, Bortezomib, Asunaprevir, Ritonavir demonstrated its effectiveness for SARS-Cov-2 in multiple in-vitro studies [51]–[56]. Moreover, Chloroquine has been shown as an effective inhibitor in [47], [58]. Nevertheless, these clinical studies depend on prior experienced knowledge that enables the selection of specific drugs that have some inhibitory possessions on similar coronaviruses. In contrast, the proposed architecture was pre-trained on several binding databases without domain experience.

*Toxicity Information:* For the Cilostazol (100 mg twice/daily) the signs of an acute overdose can be a severe headache, diarrhea, hypotension, tachycardia, and a potential increase in heart rate. The public side effects of Baricitinib (2 mg/day) are herpes simplex and zoster infections, cholesterol and creatinine elevations, neutropenia, fatigue, diarrhea nausea, and symptoms of upper respiratory tract infection. Besides, side effects of both of the Itraconazole and Fluconazole (100-400 mg/day) embrace headache, vomiting, nausea, and it exhibits rare yet serious cases of serious hepatic toxicity [4]. Quercetin (500-1000 mg/day) has similar toxicity as Itraconazole plus abdominal discomfort however it did not report any Hepatic toxicity [57]. The Rabeprazole (120 mg/day) shows some rare side effects including hypersensitivity reactions, hypomagnesemia, bone fractures for lung in case of lung use, lupus erythematosus, and acute interstitial nephritis. Further, Grazoprevir (100 mg/day) has been reported to cause mild effects including fatigue, headache and nausea; and multiple hypersensitivity reactions have been reported for Abacavir sulfate (300 mg/day) which occurred in association with anaphylaxis, liver and renal failure, hypotension, fever, rash, fatigue, GI symptoms such as nausea, vomiting, diarrhea, and abdominal pain. The recommended dosage of Bortezomib (1.3 mg/m^2^) differs by indication, tolerance, and hepatic function, and it shows fatal outcomes when the patient follows the administration of more than twice the recommended therapeutic dose; and include the acute onset of symptomatic hypotension and thrombocytopenia [52]. Further, Extrahepatic manifestations due to Rifampin (60 mg/day) hepatotoxicity such as fever, rash, arthralgias, edema, and eosinophilia are uncommon as is autoantibody formation. Several contrary effects of either Lopinavir (800 mg/day) or Ritonavir (50-100 mg twice /day) may arise including hepatotoxicity, pancreatitis, and hyperlipidemia and lipodystrophy [55], [56]. The tetraethylene glycol (2000 mg/day) caused minimal skin irritation and was not a skin sensitizer when tested in humans. Also, an overdose of Adenosine monophosphate (500 mg/day) could cause local erythema, slight flushing, dizziness, diuresis, and palpitation.

Moreover, the chloroquine (500 mg/week) overdose can trigger an acute attack with drowsiness, visual disturbances, and serum aminotransferase elevations, occasionally resulting in jaundice [47]. Hydroxychloroquine [58] (400 mg/day) does not cause this reaction and appears to have partial beneficial effects in porphyria with an exception for patients with porphyria cutanea tarda; where relatively high doses can trigger an acute hepatic injury with sudden onset of fever and marked serum enzyme elevations with increased excretion of porphyrins. Furthermore, the overdose of Atazanavir (400 mg/day) can cause several forms of liver injury including transient serum enzyme elevations, indirect hyperbilirubinemia, idiosyncratic acute liver injury, and exacerbation of underlying chronic viral hepatitis. Meanwhile, hepatic artery thrombosis has been stated to be known with sirolimus (2 mg/day) therapy after liver transplantation, but this suggestion is still controversial. Also, an overdose of Ivermectin (15 mg/day) could cause some adverse effects including muscle or joint pain, dizziness, fever, headache, skin rash, and fast heartbeat [48]. Rifaximin (550 mg/day) shows some adverse effects include peripheral edema, muscle spasms, and gastrointestinal upset. Yet there is no evidence that an overdose can cause liver injury. The Digoxin (0.1 mg/day) toxicity may be established by indications of nausea, vomiting, visual changes, in addition to arrhythmia. Older age, lower body weight, and decreased renal function or electrolyte abnormalities lead to an increased risk of digoxin toxicity [4].

## VI. Limitations of This Study

Despite the superiority of the proposed DeepH-DTA, it still suffers from some shortcomings that limit realizing the most optimal performance. First, the marginal improvement in CI measure (0.025, 0.026) could be reasoned by the fact that the model considers learning the representations of proteins sequences and drug molecules separately, then merges these representations for final decisions. This could be handled by learning the interaction patterns between proteins sequences and drug molecules. Second, the DeepH-DTA the semantic representation of input sequences that have been shown effective in many sequential data. Transformer models [63], [64] could be employed to generate more informative sequential data representation. Third, the execution time required by HGAT is high, as presented in Table 5. Fourth, some of the predictions for SARS-Cov-2 also need to be confirmed in vitro, in vivo, and in an inclusive series of scientific trials for effectiveness and safety.

## VII. Managerial Implications

This section provides a summary of the results and how our model could be useful in real-life situations. In this study, we introduce an efficient and applicable deep learning approach (DeepH-DTA) drug-target affinity prediction that is able to support clinical staff to discover the most effective inhibitor against newly discovered diseases like COVID-19. The major advantages of the proposed architecture are its capability to exploit the topological and sequential representation of drug molecules. Second, it is not restricted to the specific data used in this paper. In other words, it is possible to apply deepH-DTA to various drug repurposing problems as shown with COVID-19 data.

## VIII. Conclusion and Future Directions

We introduce a novel deep-learning framework for predicting DT binding affinity using target protein sequences and various heterogeneity drugs. We use squeezed-excited dense convolutional networks to capture hidden representations of proteins sequence. We adopt a modified HGAT network for topological modeling information of heterogeneous chemical molecules, while BConvLSTM exploited the spatio-sequential description of SMILES encoded molecules. The generated representations are concatenated and passed for final FCL, where the final affinity value is estimated. From comparative experiments with recent approaches, we conclude that our model outperforms the state-of-the-art approaches. However, our model construction did not show binding locations within raw sequence data, which provide clinically interpretable results. Moreover, we applied our model for estimating the binding affinities between SARS-CoV-2 and FDA drugs for predicting optimal antiviral inhibitors, and we find that some of our models predicted output had been approved for studies or clinical trials.

In future work, we are intended to extend our model to negative samples of DT pairs in binary classification-based DTI. Further, we intend to exploit the generative approach along with heterogeneous graph networks for drug repurposing. Additionally, we will adapt our approach to addressing multi-target interactions and we will explore recent advances in the language model for generating contextual embedding for protein sequences.
